# Comparative genomic analysis of human infective *Trypanosoma cruzi* lineages with the bat-restricted subspecies *T*. *cruzi marinkellei*

**DOI:** 10.1186/1471-2164-13-531

**Published:** 2012-10-05

**Authors:** Oscar Franzén, Carlos Talavera-López, Stephen Ochaya, Claire E Butler, Louisa A Messenger, Michael D Lewis, Martin S Llewellyn, Cornelis J Marinkelle, Kevin M Tyler, Michael A Miles, Björn Andersson

**Affiliations:** 1 Department of Cell and Molecular Biology, Karolinska Institutet, Box 285, Stockholm, SE, 171 77, Sweden; 2 Norwich Medical School, University of East Anglia, Norwich, Norfolk, NR4 7TJ, United Kingdom; 3 Department of Pathogen Molecular Biology, Faculty of Infectious and Tropical Diseases, London School of Hygiene and Tropical Medicine, Keppel Street, London, United Kingdom; 4 Centro de Investigaciones en Microbiología y Parasitología Tropical, Universidad de los Andes, Santafé de Bogotá, Colombia

## Abstract

**Background:**

*Trypanosoma cruzi marinkellei* is a bat-associated parasite of the subgenus *Schizotrypanum* and it is regarded as a *T*. *cruzi* subspecies. Here we report a draft genome sequence of *T*. *c*. *marinkellei* and comparison with *T*. *c*. *cruzi*. Our aims were to identify unique sequences and genomic features, which may relate to their distinct niches.

**Results:**

The *T*. *c*. *marinkellei* genome was found to be ~11% smaller than that of the human-derived parasite *T*. *c*. *cruzi* Sylvio X10. The genome size difference was attributed to copy number variation of coding and non-coding sequences. The sequence divergence in coding regions was ~7.5% between *T*. *c*. *marinkellei* and *T*. *c*. *cruzi* Sylvio X10. A unique acetyltransferase gene was identified in *T*. *c*. *marinkellei*, representing an example of a horizontal gene transfer from eukaryote to eukaryote. Six of eight examined gene families were expanded in *T*. *c*. *cruzi* Sylvio X10. The DGF gene family was expanded in *T*. *c*. *marinkellei*. *T*. *c*. *cruzi* Sylvio X10 contained ~1.5 fold more sequences related to VIPER and L1Tc elements. Experimental infections of mammalian cell lines indicated that *T*. *c*. *marinkellei* has the capacity to invade non-bat cells and undergo intracellular replication.

**Conclusions:**

Several unique sequences were identified in the comparison, including a potential subspecies-specific gene acquisition in *T*. *c*. *marinkellei*. The identified differences reflect the distinct evolutionary trajectories of these parasites and represent targets for functional investigation.

## Background

The subgenus *Schizotrypanum* harbors the type species *Trypanosoma cruzi*, which is the causative agent of Chagas disease in humans. Other members of the *Schizotrypanum* subgenus are often referred to as *T*. *cruzi*-like species as they are morphologically similar or indistinguishable from *T*. *cruzi*[1]. With the exception of the human infecting parasite, members of *Schizotrypanum* are restricted to bats (order *Chiroptera*) and occur in high prevalence among bats in Latin America and elsewhere in the world [1-4]. There is no evidence that *T*. *cruzi*-like parasites are harmful to bats, although this may reflect a paucity of data. Most infected bats are insectivorous and infection is thought to take place either through ingestion of infected arthropods or via stercorarian transmission from bat-feeding bugs [5,6]. The genetic diversity of *T*. *cruzi*-like species and their evolutionary relationships are yet to be determined.

*Trypanosoma cruzi marinkellei* is a bat-associated subspecies of *T*. *cruzi*[1]. The human infective parasite *T*. *cruzi* should accordingly be referred to as the nominate subspecies *T*. *cruzi cruzi* (*T*. *c*. *cruzi*) [1]. *T*. *c*. *marinkellei* is prevalent among bats in Central and South America, which are its only known mammalian hosts [1,5]. It differs from *T*. *c*. *cruzi* in terms of isoenzyme electrophoresis patterns and buoyant DNA densities. *T*. *c*. *marinkellei* does not infect immunocompetent mice [1,5], nor does it provide immunological protection against challenge with *T*. *c*. *cruzi*[1], suggesting that the infection is characterized by distinct antigenic profiles. Sequence-based phylogenies have confirmed a relatively close relationship with *T*. *c*. *cruzi*[5,7-9] and estimated the divergence time at ~6.5-8.5 MYA [10-12]. Cavazzana *et al*. reported that *T*. *c*. *marinkellei* was associated with phyllostomid species (insectivorous, frugivorous, carnivorous and haematophagous bats) [5] and transmission is thought to occur when triatomine bugs of the genus *Cavernicola* feed on bats [13]. However, the natural transmission cycle among bats is not well characterized and there might be other vectors or direct transmission mechanisms. Some genetic substructure within the *T*. *c*. *marinkellei* population has been reported [14], but the strength of correlation between parasite lineage and host remains to be defined. Moreover, bat-restricted parasites are of evolutionary interest, since it has been proposed that *T*. *c*. *cruzi* may have originated from an ancestral bat-lineage that jumped into terrestrial mammals [15]. The present day human lineage, *T*. *c*. *cruzi*, has been in contact with humans for no more than 10,000 to 30,000 years, which is the period of human presence in the Americas [16].

*T*. *c*. *cruzi* strains are currently sorted into six lineages or discrete typing units (DTUs), which illustrate the genetic diversity of this parasite [17]. Several strains have to date been subjected to genome sequencing, among these are CL Brener and Sylvio X10. The CL Brener strain was selected for the original genome project and belongs to DTU VI. The size of the CL Brener genome was ~110 Mb and it was assembled mostly with Sanger paired-end reads. The CL Brener strain was shown to be a genetic hybrid of two diverged haplotypes named Esmeraldo-like and non-Esmeraldo-like [18]. The hybrid and repetitive nature of this genome complicated sequence assembly and finishing, leaving the genome in many gaped scaffolds and contigs. Weatherly *et al*. later compiled scaffolds into more complete chromosome-wide sequences [19]. Second-generation sequencing facilitates more cost-effective and rapid sequencing efforts. Recently, 454-sequencing was applied on the genome of the DTU I strain Sylvio X10 [20], revealing a slightly smaller but still repeat-rich genome.

Little is known about genomic variation among organisms within the *Schizotrypanum* genus. Genomic insights can provide information on evolutionary adaptation of these parasites, as well as being useful for advancing population genetics. Thus, exploring genomic diversity could reveal important genetic and biological characteristics, and potentially clues as to how these parasites relate to the human disease. Here we describe the genome of *T*. *c*. *marinkellei* B7, a bat-associated parasite originally isolated from a colony of the pale spear-nosed bat *Phyllostomus discolor* roosting in a hollow tree [1]. The parasite was isolated in São Felipe, Bahia state, Brazil in 1974 and has since then been stored under cryogenic conditions with occasional short periods of *in vitro* cultivation. We combined Roche/454 and Illumina sequencing to generate a draft genome sequence of *T*. *c*. *marinkellei*. This is the first whole genome analysis of a *T*. *c*. *cruzi*-like species that is not associated with human infections. In addition, we also report re-assembly and re-annotation of the human infective strain *T*. *c*. *cruzi* Sylvio X10 [20], a commonly used reference strain of *T*. *c*. *cruzi* I [21], using additional sequence data.

The comparative analyses with *T*. *c*. *cruzi* revealed that the genomes contain the same repertoire of housekeeping genes. Moreover, *T*. *c*. *marinkellei* contains an additional gene that appears to be an example of recent horizontal gene transfer. In addition, the genomes also exhibit copy number variation and diversification of gene families, which potentially give rise to a large number of strain-specific protein isoforms.

## Results and discussion

### Sequencing and Assembly of *T*. *c*. *marinkellei* and *T*. *c*. *cruzi* Sylvio X10

In the text, we refer to *Trypanosoma cruzi marinkellei* as *Tcm*, *Trypanosoma cruzi cruzi* Sylvio X10 as *Tcc* X10 and *Trypanosoma cruzi cruzi* CL Brener as *Tcc* CLBR. Genomic sequence reads were generated from *Tcm* and *Tcc* X10 using 454 and Illumina sequencing (Table 1). 454 sequencing (single end; long reads) was performed on genomic DNA from *Tcm*, which produced sequence reads with an average length of ~357 nt. The 454 data from *Tcc* X10 was the same as previously described [20]. In addition, one ~2 kb insert library (2×100-nt reads) was prepared for *Tcm* and *Tcc* X10 respectively, using a modified version of the Illumina mate-pair protocol (Materials and Methods). The modified Illumina protocol was chosen to enable 100 nt read lengths, as Illumina does not recommend its own protocol for mate-pair sequencing with read lengths >36 nt. This generated 71,948,029 and 84,638,048 read-pairs from *Tcm* and *Tcc* X10 respectively. Not all read-pairs translated to the expected insert size of ~2 kb. Long insert libraries often contain a significant proportion of short insert fragments (corresponding to paired-end reads). Most often this is due to non-optimal biotin enrichment causing some fragments not to circularize and therefore become sequenced with much shorter insert. We determined the number of true mate-pairs from the obtained data using an R-script previously published by Van Nieuwerburgh *et al*. [22]. The script determines the location of the LoxP linker sequence in the read, and then uses this information to classify read-pairs as true mate-pairs, paired-end, single-end or linker-negative. True mate-pairs should contain the LoxP sequence close to the 3^′^ end in at least one read, indicating that circularization has taken place. In our data, 32% (23,055,208/71,948,029) and 34% (28,781,049/84,638,048) of the read-pairs were classified as true mate-pairs from *Tcm* and *Tcc* X10 respectively (LoxP sequence close to the 3^′^ end in at least one of the reads). 38% (27,890,116/71,948,029) and 35% (30,076,419/84,638,048) read-pairs were classified as paired-end from *Tcm* and *Tcc* X10 respectively. The remaining read-pairs were either unpaired or LoxP-negative, meaning that the linker was present in the unsequenced part of the fragment or that the fragment did not contain a linker. Hence, despite an improved protocol, a substantial number of paired-end and single-end reads were obtained. The causes of this has previously been discussed [22].

**Table 1:** Raw sequence data

	***T***. ***c***. ***marinkellei***		***T***. ***c***. ***cruzi *Sylvio X10**	
	**454**^** a**^	**Illumina**^** b**^	**454**^** a**^	**Illumina**^** b**^
# **reads** (**10**^**6**^)	1.3	23.0	1.3	28.7
# **nt** (**10**^**9**^) ^**c**^	0.47	35.6	0.52	44.3
**Average read length** (**nt**) ^**d**^	357	77	393	77
~ **Coverage**^** e**^	12	91	9	103

^a^ Single end 454 reads.^b^ No. Read-pairs (true mate-paired reads after adapter trimming).^c^ Billion nucleotides.^d^ The average read length (after adapter trimming).^e^ The theoretical genome coverage based on known genome sizes and the number of sequenced nucleotides.

**Figure 1:**
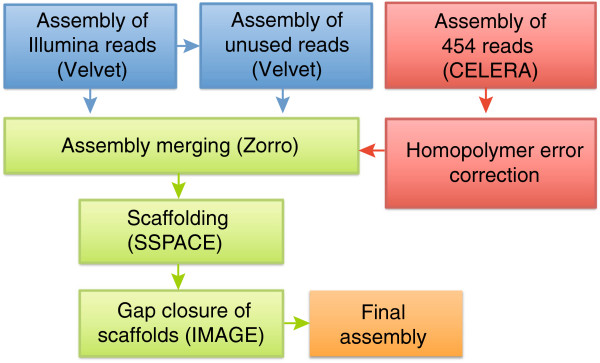
**Schematic overview of the sequence assembly.** Schematic overview of the genome assembly steps. Illumina reads were assembled into contigs with Velvet. Unused reads were extracted and used for a second Velvet assembly with a different kmer length. 454 reads were assembled with CELERA. The 454-assembly was then subjected to homopolymer error correction with Illumina reads. The Illumina and 454 assemblies were merged into a non-redundant assembly using the Zorro pipeline. The assembly was then subjected to scaffolding using SSPACE and physical distance information. The final step involved gap closure with the IMAGE pipeline.

The 454 and Illumina data were subsequently assembled (Figure 1). In order to take platform dependent sequencing artifacts into consideration, 454 and Illumina reads were assembled separately using different assembly programs (Figure 1; Table 2; Materials and Methods). Insertion-deletion errors in the 454 assemblies were identified and corrected using alignments with Illumina reads, which corrected 12,358 and 7,277 positions of *Tcm* and *Tcc* X10 respectively. The most common error was one or two missing bases (~90% of the corrected positions). The resulting assemblies were subsequently merged into a non-redundant assembly. Distance information from mate-pair reads was used to arrange contigs into scaffolds. Where possible, the distance between two adjacent contigs in a scaffold was inferred by comparison with *Tcc* CLBR, i.e. if two contigs flanking each side of a gap could be aligned with one of the CL Brener haplotypes, then the approximate gap length could be inferred from CL Brener. As a final assembly step, both *Tcm* and *Tcc* X10 were subjected to gap closure using the IMAGE pipeline [23] and the sorted paired-end reads (see above). Prior to feeding scaffolds into IMAGE, paired-end reads were quality filtered. IMAGE uses iterative mapping of reads to contig ends, followed by local assembly and alignment to close gaps and extend contigs. Eight IMAGE iterations were completed for each genome, which improved each assembly by adding 653,655 (*Tcm*) and 534,614 (*Tcc* X10) base pairs, which closed 261 and 171 gaps and extended 2,426 and 2,510 contig ends from *Tcm* and *Tcc* X10 respectively.

**Table 2:** Genome assembly statistics and summary

**Genome**	**Step**	**Software**	**Size**^** a**^	# **contigs**^** b**^	# **scaffolds**	**Average length**^** c**^	**N50**^** d**^	**N90**^** e**^
***Tcm***	454 assembly	CELERA	37.3	30,737	-	1,216	1,670	539
	Illumina assembly	Velvet (kmer 43)	16.7	9,247	-	1,813	2,378	851
	Assembly of non-assembled Illumina reads	Velvet (kmer 53)	1.17	2,094	-	562	536	418
	Assembly merging	Zorro	33.5	24,799 ^f^	-	1,353	2,218	549
	Scaffolding	SSPACE	38.8	23,813 ^f^	1,835	2,296	25,044	576
	Gap closure	IMAGE	38.6	23,000	1,774	2,302	25,781	583
***Tcc *X10**	454 assembly	CELERA	41.8	33,686	-	1,243	1,516	549
	Illumina assembly	Velvet (kmer 43)	17.0	8,523	-	1,997	2,742	904
	Assembly of non-assembled Illumina reads	Velvet (kmer 53)	1.14	2,116	-	543	523	416
	Assembly merging	Zorro	38.0	28,389 ^f^	-	1,339	1,869	560
	Scaffolding	SSPACE	43.7	27,605 ^f^	2,476	2,162	14,067	589
	Gap closure	IMAGE	43.4	26,889	2,423	2,158	14,516	592

^a^ The length when sequences are combined (Mb).

^b^ The number of contigs/scaffolds.

^c^ The average contig length (bp). For the SSPACE row, this refers to the average scaffold length.

^d^ The length N for which half of all bases are in a sequence of this length or longer.

^e^ The length N for which 90% of all bases are in a sequence of this length or longer.

^f^ Contigs >500 bp.

The combined assembly lengths were 38.6 Mb and 43.4 Mb for *Tcm* and *Tcc* X10 respectively. The assembly size of *Tcc* X10 was very similar to our previous estimate from extrapolation of unassembled data [20] and flow cytometry [24]. Flow cytometry analysis estimated the haploid size of *Tcm* to ~39 Mb (Additional file 1: Figure S1), which was close to the *in silico* assembly length. Thus, assembly sizes were consistent with experimental measurements. Moreover, this confirmed that the *Tcm* genome was ~4.8 Mb smaller than that of *Tcc* X10. The percentage of assembled bases in each assembly was very similar: *Tcm* 88.6% (34.2 Mb/38.6 Mb); *Tcc* X10 88.7% (38.5 Mb/43.4 Mb). We analyzed 29,422 unused 454 reads of *Tcm* with RepeatMasker, which identified 13,108 reads corresponding to kinetoplastid sequences. The remaining reads were analyzed with BLAST, showing them to correspond multicopy genes or other repeats.

868 (*Tcm*) and 987 (*Tcc* X10) scaffolds were longer than 5 kb, which corresponded to 25.7 and 26.8 Mb (including gaps). The longest scaffolds were 335 kb (*Tcm*) and 384 kb (*Tcc* X10). Some 200 gaps could be closed from the apparent overlap of adjacent contigs. Compared with 454 reads alone, addition of mated reads provided longer contigs and scaffolds, corrected 454 sequence errors and allowed accurate estimation of genome heterozygosity and copy number variation.

### Comparison of heterozygosity and multicopy genes

**Figure 2:**
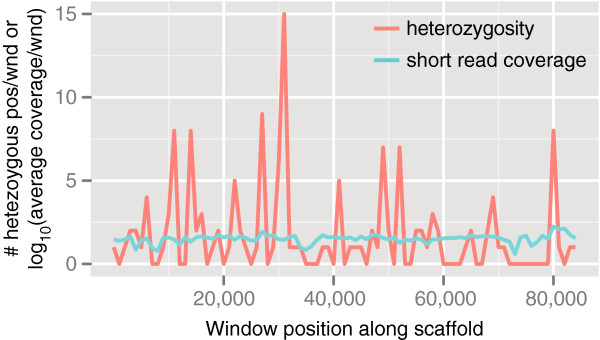
**Distribution of heterozygosity along a genomic segment of *T***. ***c***. ***marinkellei *.** Distribution of heterozygosity and sequence coverage along scaffold 143 of *T*. *c*. *marinkellei* B7. Heterozygosity was counted in non-overlapping sliding windows of 1000 bp (red line). Coverage is shown as the log_10_-scaled average coverage of the 1000 bp window (turquoise line). The x-axis shows the start position of the window along the sequence and the y-axis shows the number of heterozygous nucleotide positions per window or log_10_-scaled average coverage of the window.

The level of heterozygosity among populations of medically important trypanosomes is likely to reflect the impact of key evolutionary processes such as gene conversion and genetic exchange. In the present study we estimated the amount of heterozygosity in *Tcm* and *Tcc* X10 by aligning Illumina and 454 reads back to the assemblies and subsequently identifying high quality mismatches between the consensus sequence and aligned reads. In order to increase the confidence, only nucleotide positions with 10 to 80X coverage were included and contigs shorter than 5 kb were ignored. This resulted in 19,015,919 and 20,468,447 positions of *Tcm* and *Tcc* X10 that permitted analysis, which represented 49.2% (19.0 Mb/38.6 Mb) and 47.0% (20.4 Mb/43.4 Mb) of each genome respectively. Furthermore, a mismatch had to be supported by at least 9 reads in order to call the position heterozygous. The search identified 37,894 positions of *Tcm* and 46,001 positions of *Tcc* X10 that were heterozygous. Taken together, genome heterozygosity levels of *Tcm* and *Tcc* X10 were ~0.19% (37,894 bp/19,015,919 bp) and ~0.22% (46,001 bp/20,468,447 bp), of which 38.8% (14,712 bp/37,894 bp) and 42.4% (19,513 bp/46,001 bp) were located in protein-coding genes. 7,976 and 10,596 heterozygous positions of *Tcm* and *Tcc* X10 were located at non-synonymous sites. Gene Ontology analysis was performed on genes containing at least one polymorphism at a non-synonymous site, resulting in two significantly enriched categories (*p*<0.05): GO:0009451 (RNA modification) and GO:0009982 (pseudouridine synthase activity). Overall, the estimated level of heterozygosity of *Tcc* X10 was slightly higher than previously reported [20], likely due to the increased sequence depth in the present study. In order to identify regions with higher density of heterozygosity, i.e. clustering of heterozygous sites, we counted the number of heterozygous positions inside 1,000 bp windows. This indicated that heterozygosity often, but not exclusively, was located in clusters (Figure 2). In conclusion, heterozygosity of the *Tcm* and *Tcc* X10 were ~0.19% and ~0.22%, with some regions exhibiting higher than average heterozygosity. In contrast, the heterozygosity level of *Tcc* CLBR was ~1 to 4% (since it is a hybrid). In comparison to other kinetoplastids, the heterozygosity level is similar to that of *Leishmania braziliensis* but higher than *L*. *major* and *L*. *infantum*[25]. The generally low levels of heterozygosity found in many protozoans is difficult to explain in terms of a strictly clonal propagation model [10]. Such organisms would be expected to observe extensive divergence of homologous genomic copies, which is the case for bdelloid rotifers [26]. In perspective, the B lineage of the human parasite *Giardia intestinalis* exhibits relatively high heterozygosity (~0.5%) [27] whereas A and E lineages exhibit low heterozygosity (~0.01%) [28]. The genome of the free-living amoeboflagellate *Naegleria gruberi* was described as mosaic of homozygous and heterozygous regions, with an average polymorphism rate of 0.58% [29]. Interestingly, asexual lineages of *Daphnia* exhibit low levels of allelic divergence and appear to employ ameiotic recombination to eliminate heterozygosity faster than it accumulates [30]. The mechanism for maintaining low heterozygosity in trypanosomatids remains unknown, but could involve cryptic sexuality, frequent local gene conversion or chromosome-wide conversion. The former can be evaluated via an assessment of population-level inter-locus linkage disequilibrium. Nevertheless, descriptive data may not be sufficient to explain the causes of this phenomenon.

*T*. *c*. *cruzi* contain several highly expanded and complex gene families [31,32], comprised of transcribed genes and pseudogenes. Several of these families have been reported to vary in copy number between strains [18,20,33-35]. In the present study we compared gene family content between *Tcm* and *Tcc* X10 using the depth of aligned short reads. Initially, repeat boundaries were determined using RepeatMasker. Subsequently, the percentages of reads mapping to repeat families were calculated (Table 3). The statistical significance was assessed in the following way: an empirical distribution of genome-wide read-depth differences was established using regions of homology between *Tcm* and *Tcc* X10 (Additional file 2: Figure S2). The software promer was used to find homologous regions. In each homologous region, the percentage read coverage was calculated for *Tcm* and *Tcc* X10. These numbers were then corrected for the genome size and the coverage difference for each homologous region was computed. 17,290 regions were included, with mean 1.380393e-07 and standard deviation 5.83481e-07. The logspline function of the R package with the same name was used to fit a smoothed density curve to the data, and the plogspline function was used to determine *p*-values. Six out of eight examined gene families were expanded in *Tcc* X10: *trans*-sialidase; mucin-associated surface protein; retrotransposon hot spot protein; TcMUC mucin; ABC Transporter; and RNA binding protein. On the contrary, GP63 and dispersed gene family 1 (DGF) were contracted in *Tcc* X10. The observation that DGF was contracted in *Tcc* X10 was consistent with previous data when *Tcc* X10 was compared with *Tcc* CLBR [20], which suggests a recent loss of DGF-related sequences in the lineage leading to *Tcc* X10. Further examination of several DTU I strains may resolve if this is a general feature of this lineage. It is clear that at least part of the genome size difference can be attributed to expansion and/or contraction of these gene families. We performed a closer examination of the TcMUCII mucin gene family. TcMUCII mucin genes of the same genome were frequently found to be too different to align. We constructed entropy plots from alignment positions that were deemed as accurate, which revealed, as expected that 5′ and 3′ termini were more conserved and the internal parts of these genes were hypervariable (Additional file 3: Figure S3).

**Table 3:** Comparison of gene family content

	***T.c. marinkellei***		***T.c. cruzi *Sylvio X10**		
**Gene family**^** a**^	**Size in assembly**^** b**^	**% Short reads**^** c**^	**Size in assembly**^** b**^	**% Short reads**^** c**^	**SE**^** d**^
**DGF**	2,129,983 (6.22 %)	3.433	1,265,650 (3.28 %)	1.324	*Tcm*
**TS**	2,109,163 (6.16 %)	6.291	2,953,602 (7.65 %)	6.298	*Tcc* X10
**MASP**	540,360 (1.58 %)	1.317	727,537 (1.88 %)	1.434	*Tcc* X10
**RHS**	521,665 (1.52 %)	2.234	1,314,589 (3.41 %)	2.915	*Tcc* X10
**GP63**	452,732 (1.32 %)	1.229	514,422 (1.33 %)	0.898	*Tcm*
**TcMUC mucin**	273,890 (0.80 %)	0.557	334,544 (0.87 %)	0.515	*Tcc* X10
**ABC**	37,490 (0.11 %)	0.124	42,072 (0.11 %)	0.162	*Tcc* X10
**RBP**	25,946 (0.08 %)	0.080	26,732 (0.07 %)	0.074	*Tcc* X10

^a^ Gene family abbreviations: DGF=Dispersed Gene Family, TS=*trans*-sialidase, MASP=Mucin-associated surface protein, GP63=Surface protease, RHS=Retrotransposon Hot Spot protein, ABC=ABC Transporter, RBP=RNA Binding Protein.

^b^ The combined number of base pairs of this gene family that was identified in the assembly. Sequences were identified using RepeatMasker and a repeat library of coding sequences from the *Tcc* CLBR genome. These numbers include partial coding sequences. The number inside parenthesis refers to the percentage of total assembly size.

^c^ The percentage of short reads that mapped to these features.

^d^ SE=Significantly Enriched. Refers to if one genome contained significantly more of this gene family. The significance was determined from an empirical distribution of read depth differences from homologous regions of *Tcm* and *Tcc* X10, corrected for genome size. The empirical distribution was used to calculate a *p*-value.

### Kinetoplastid DNA (maxicircle)

The mitochondrial genomes (maxicircles) of *T*. *c*. *cruzi* strains X10 (DTU I), Esmeraldo (DTU II) and CLBR (DTU VI) have been sequenced, and have provided insights into the structure and organization of kinetoplastid DNA of these strains [36]. The *T*. *c*. *marinkellei* maxicircle was identified as a 20,037 bp contig from the 454 assembly. The length of this sequence was slightly longer (~ 5 kb) than those previously reported, and the difference was attributed to variability in the repetitive region. The coding region of the *Tcm* maxicircle was syntenic with the coding regions of the three complete *T*. *c*. *cruzi* maxicircle genomes, beginning with the 12S rRNA gene and ending with the *ND5* gene. The lengths of the individual genes within the *Tcm* maxicircle coding region were comparable to those of the three *T*. *c*. *cruzi* strains (Additional file 4: Table S1). The length of the complete maxicircle coding region (beginning at 12S rRNA and ending after *ND5*) for *Tcm* was 15,438 bp and began after 4,599 bp of non-coding sequence. With respect to coding sequences, the average maxicircle nucleotide identity between *Tcm* and *Tcc* X10 was (mean ± sd): 85.12% ± 6.1, between *Tcm* and *Tcc* CLBR was 85.4% ± 6.2 and between *Tcm* and *Tcc* Esmeraldo was 85.3% ± 6.1 (Additional file 4: Table S1). Phylogenetic reconstruction of the maxicircles from *Tcm*, *Tcc* X10, *Tcc* CLBR and *Tcc* Esmeraldo confirmed that the *Tcm* maxicircle was slightly closer to *Tcc* Esmeraldo than *Tcc* X10/CLBR (Additional file 5: Figure S4). The topology of the tree suggests that the Esmeraldo maxicircle might represent the ancestral maxicircle lineage of *T*. *c*. *cruzi*.

The consensus maxicircle genome sequence is derived from the predominant nucleotide present across multiple read alignments at each position. However, this criterion disregards low abundance single nucleotide polymorphisms (SNPs) and therefore masks minor maxicircle haplotypes (heteroplasmy), which has previously been reported from *Tcc* X10 [37]. Illumina reads were used to assess the presence/absence of minor *Tcm* maxicircle haplotypes. In total, this identified 19,821 reads that aligned to the *Tcm* maxicircle. Low levels of heteroplasmy were observed in the *Tcm* maxicircle protein-coding region. Twenty SNPs were identified among four genes (*ND8*, *MURF1*, *COI* and *ND3*) and one intergenic region (between *CR4* and *ND4*). Average read depth for each SNP site was 47. At heterozygous sites, the minor nucleotide was present among an average of 9.5% (± 3.3%) of reads. All SNPs were bi-variable except for at two intergenic positions, where two minor nucleotides were present. These observations imply the occurrence of at least two minor mitochondrial haplotypes.

### Gene content analysis and comparison

**Figure 3:**
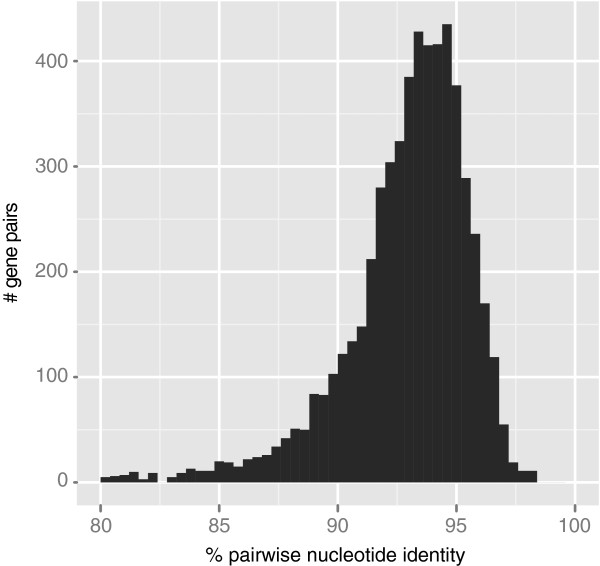
**Histogram of pairwise nucleotide identities between orthologous genes.** Histogram of pairwise nucleotide identities between orthologs of *T*. *c*. *marinkellei* B7 and *T*. *c*. *cruzi* CL Brener non-Esmeraldo-like haplotype. 5618 orthologs were included in the comparison, for which the average nucleotide identity was 92.6% ± 3.3 (*Tcm* vs *Tcc* CLBR non-Esm). The genes included in this analysis mainly comprised the non-repetitive component of these genomes. Orthologs were defined as the best reciprocal BLASTp hit between the genomes. Nucleotide sequences were aligned with ClustalW version 2.1. Mismatches (single nucleotide polymorphisms) within each alignment were identified and counted using a Perl script. Pairwise orthologs with lower identity than 80% were excluded from the analysis.

The *Tcm* and *Tcc* X10 genomes were annotated using a semi-automatic strategy, which relied on the previous annotation of the reference genome *Tcc* CLBR [18]. Gene models were transferred from *Tcc* CLBR to *Tcm* and *Tcc* X10 using Perl scripts, reciprocal BLASTp searches together with positional information (Materials and Methods). In addition, gene prediction was performed and gene models were kept if one or more of the following criteria were satisfied: (*i*) the gene was conserved in a syntenic position in *Tcc* CLBR; (*ii*) the gene shared homology with one or more gene families in *Tcc* CLBR; and (*iii*) the gene was longer than 250 amino acids. Gene models with complete overlap with another gene were discarded. The final annotations were manually inspected and refined with the Artemis Comparison Tool [38]. After this procedure, the genome sequences contained 10,342 (*Tcm*) and 11,112 (*Tcc* X10) protein coding gene annotations, of which 60.5% (6,267/10,342) and 57.7% (6,416/11,112) were syntenic with *Tcc* CLBR, *Tcm* and *Tcc* X10 respectively. With respect to coding sequences, the average nucleotide identity between *Tcm* and *Tcc* X10 was 92.5% ± 3.2 (Figure 3). When *Tcm* was compared with *Tcc* CLBR Esm and non-Esm the average nucleotide identity was 92.8% ± 3.4 and 92.6% ± 3.2. These identities are based on a total of 6,283 (*Tcm*:*Tcc* X10), 5,441 (*Tcm*:*Tcc* CLBR Esm) and 5,617 (*Tcm*:*Tcc* CLBR non-Esm) orthologous gene pairs. ~86% (14,997/17,332) of the analyzed ortholog pairs had a nucleotide identity of 90% or higher. The ratio of non-synonymous and synonymous nucleotide substitutions (ω=dN/dS) was 0.31 ± 0.21 in average (*Tcm* versus *Tcc* CLBR non-Esm), indicating as expected that most genes were under purifying (stabilizing) selection. A total of 69 genes showed ω values > 1.1, indicating positive selection (Additional file 6: Table S2).

In order to identify isolate-specific genes, we compared the gene complements of the three genomes (*Tcm*, *Tcc* X10 and *Tcc* CLBR) using BLAST. Initially, the predicted proteomes of *Tcm* and *Tcc* X10 were queried with BLASTp against the predicted proteome of *Tcc* CLBR using the E-value threshold 1e-20. This resulted in 237 (*Tcm*) and 290 (*Tcc* X10) proteins longer than 250 amino acids that were not found in the *Tcc* CLBR proteome. These protein sequences were queried using tBLASTn against the *Tcc* CLBR genome to exclude the possibility that these putative genes were present as non-annotated open reading frames, using the same E-value threshold. This decreased the number of hits to 22 (*Tcm*) and 3 (*Tcc* X10). The composition of the 22 putative *Tcm*-specific genes were as follows: 11 TcMUCII mucin genes; 1 acetyltransferase (MOQ_006101); 5 putative genes with weak hits to microbial sequences (MOQ_006053, MOQ_007485, MOQ_009774, MOQ_006631, MOQ_003304); and 5 putative genes with no hits in public databases (MOQ_003636, MOQ_009528, MOQ_006983, MOQ_009799, MOQ_005225). For *Tcc* X10, one of the specific genes corresponded to a diverged mucin-associated surface protein (TCSYLVIO_008353). The remaining two putative genes did not show any significant hits in public databases (TCSYLVIO_011068 and TCSYLVIO_008789). Thus, the improved *Tcc* X10 genome sequence facilitated the detection of two putative *Tcc* X10-specific protein-coding genes not apparent in the earlier version. The two unknown genes were found to also be present in the previously reported draft genome sequence of *Tcc* X10 [20].

We used the same strategy to perform the reversed search, i.e. searching for genes specific for *Tcc* CLBR. This resulted in 344 and 206 protein sequences that were not found in *Tcm* and *Tcc* X10. Searches using tBLASTn towards *Tcm* and *Tcc* X10 further decreased this number to 70 and 100, and of these 52 and 21 were mucin-associated surface proteins or TcMUCII mucin. 8 (*Tcm*) and 26 (*Tcc* X10) contained low complexity repeats. The remaining 10 (*Tcm*) and 53 (*Tcc* X10) genes were queried against the raw 454 reads of *Tcm* and *Tcc* X10, which further decreased the number of *Tcc* CLBR specific genes to 3 that were not present in *Tcm* (Tc00.1047053511585.110, Tc00.1047053509525.260, Tc00.1047053510073.24). The 3 genes were uncharacterized (hypothetical). The *Tcc* CLBR-specific genes, compared with *Tcc* X10, were identical to those previously reported [20]. In conclusion, the total number of specific genes was remarkably low in relation to the number of coding sequences in these genomes. As a perspective, comparative genomics of *T*. *brucei brucei* and *T*. *brucei gambiense* did not identify any gene that could explain the ability to infect different species, despite interspecific pathological variation [39].

### A specific acetyltransferase gene in *T*. *c*. *marinkellei*

**Figure 4:**
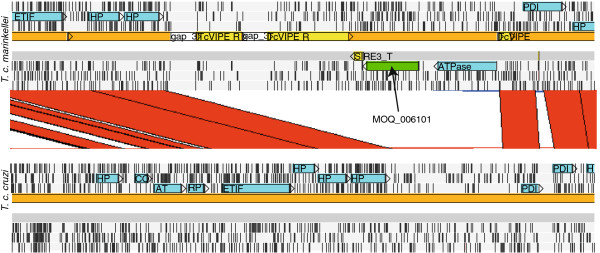
**Genomic location of the *T. c. marinkellei ***-**specific acetyltransferase gene(MOQ**_**006101).** Screenshot from Artemis Comparison Tool of a ~19 kb homologous region of *T*. *c*. *marinkellei* B7 (contig 2842) and *T*. *c*. *cruzi* CL Brener (non-Esmeraldo-like haplotype; chr 37). Vertical black lines in each frame represent stop codons. Genes with shared homology in both genomes are shown in blue and the specific *T*. *c*. *marinkellei* gene (MOQ_006101) is shown in green. Red stripes represent regions with high sequence similarity between the two genomes. Abbreviations: ETIF (eukaryotic translation initiation factor 3 subunit 8, putative); HP (hypothetical protein); PDI (protein disulfide isomerase); CO (cytochrome c oxidase subunit IX); AT (acetyltransferase); and RP (U1A small nuclear ribonucleoprotein).

As mentioned above, a 1,662 bp acetyltransferase gene (MOQ_006101) was found among the 22 unique genes in *Tcm*. This gene was identified in a single copy on scaffold 2842 and was missing in *T*. *c*. *cruzi*. Alignment of scaffold 2842 from *Tcm* with *Tcc* CLBR showed that it aligned close to the end of chromosome 37 and was flanked by VIPER elements and an ATPase gene (Figure 4). To exclude the possibility that MOQ_006101 was not properly assembled in *T*. *c*. *cruzi*, we searched raw 454/Illumina reads from *Tcc* X10 and raw Sanger reads from *Tcc* CLBR. This confirmed that MOQ_006101 was not present in these genomes. Domain searches of MOQ_006101 revealed the presence of a Cas1p domain (pfam07779, E-value=9e-66) and multiple *trans*-membrane domains. In GenBank, the best hit from protein BLAST was to the green algae *Chlamydomonas reinhardtii*, containing 44% sequence identity over 496 amino acids (E-value < 4e-125). 4 iterations of PSI-BLAST resulted in hits to various species of plants and algae. The best ten hits were to the enzyme O-acetyltransferase, displaying protein identities between ~37-39% (Table 4). This indicated that MOQ_006101 has either diverged since the transfer to *Tcm* or that it has been transferred from a species not contained in GenBank, of which the latter seems the most likely. Furthermore, transcription of MOQ_006101 was detected with reverse transcriptase quantitative polymerase chain reaction (RT-qPCR).

**Table 4:** **List of hits obtained from PSI**-**BLAST after 4 iterations querying MOQ**_**006101 against GenBank non**-**redundant database**

**Species**	**Description**	**Accession**	**CDD hit**^** a**^	**% Identity**	**BLAST E**-**value**
*Populus trichocarpa*	Predicted protein	XP_002298511.1	Cas1_AcylT	38%	0
*Arabidopsis thaliana*	Putative O-acetyltransferase	NP_568662.1	Cas1_AcylT	39%	0
*Arabidopsis thaliana*	AT5g46340/MPL12_14	AAL11600.1	Cas1_AcylT	38%	0
*Arabidopsis thaliana*	O-acetyltransferase-like protein	NP_180988.3	Cas1_AcylT	37%	0
*Populus trichocarpa*	Predicted protein	XP_002317300.1	Cas1_AcylT	37%	0
*Vitis vinifera*	CAS1 domain-containing protein 1-like	XP_002272126.2	Cas1_AcylT	38%	0
*Arabidopsis lyrata* subsp. Lyrata	O-acetyltransferase family protein	XP_002879497.1	Cas1_AcylT	37%	0
*Arabidopsis lyrata* subsp. Lyrata	Hypothetical protein	XP_002863407.1	Cas1_AcylT	39%	0
*Ricinus communis*	O-acetyltransferase, putative	XP_002519732.1	Cas1_AcylT	38%	0
*Glycine max*	CAS1 domain-containing protein 1-like	XP_003532649.1	Cas1_AcylT	38%	0

^**a**^ The best hit from the NCBI Conserved Domain Database.

**Figure 5:**
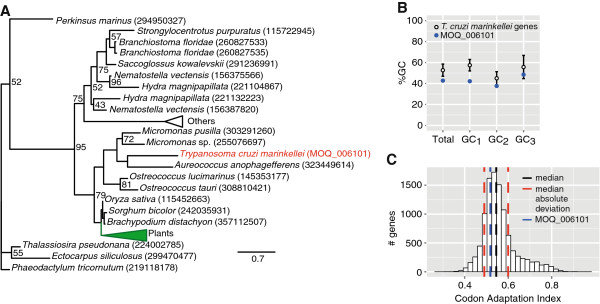
**Analyses of the *T***. ***c***. ***marinkellei ***-**specific acetyltransferase gene.** (**A**) Maximum likelihood phylogenetic tree of the *T*. *c*. *marinkellei* B7 specific gene (MOQ_006101) based on protein sequences. Phylogenetic inference was done on a protein dataset extracted with Blast Explorer (E-value <1e-40). The multiple sequence alignment was done with ClustalW version 2.1 and filtered with Gblocks. The final alignment, which the tree is based on, contained 62 columns. The phylogeny was inferred using RAxML version 7.0.4 with the PROTGAMMAJTT model and 100 bootstrap replicates. Only bootstrap values >40 are shown. Accession numbers for protein sequences are shown in parenthesis after species names. The *Tcm* gene is shown in red. (**B**) GC content analysis of MOQ_006101 in relation to all genes. Error bars represent one standard deviation. The white dot represents all genes and the blue dot represents MOQ_006101. GC_1_, GC_2_ and GC_3_ refer to the %GC content at the first, second and third codon positions. 10,342 coding sequences were included in the analysis. (**C**) Histogram of Codon Adaptation Index (CAI) for all genes in the *Tcm* genome. 43 ribosomal proteins were used as a reference for highly expressed genes. The vertical black line represents the median CAI (0.545), the two red lines represent +/− one median absolute deviation (0.0548) and the blue line represents the CAI of MOQ_006101 (0.518). CAI was calculated using emboss programs *cai* and *cusp*.

Phylogenetic reconstruction of MOQ_006101 demonstrated that the closest known homologs were from various species of algae and plants (Figure 5A), and the absence of exon-intron boundaries suggested that it was transferred as a spliced mRNA. No homologs were found in *Trypanosoma rangeli* (Edmundo C. Grisard, Personal communication), *Rhodnius prolixus* (insect vector) or *Myotis lucifugus* (a bat species). The GC content of MOQ_006101 was 42.8%, which was significantly lower than the average of 52.7% ± 5.8 (Figure 5B). The GC content of the first, second and third codon positions were 42.2%, 37.7% and 48.6%, in contrast to the global levels: 57.5% ± 5.4 (GC_1_), 45.0% ± 6.2 (GC_2_) and 55.7% ± 11.0 (GC_3_). Hence, the GC content of MOQ_006101 was unusually low in relation to the global GC content of all *Tcm* genes. In conclusion, this suggested that the nucleotide composition of MOQ_006101 was distinct compared with all other genes of the *Tcm* genome. The unusual GC content can be interpreted as an imprint from the originating genome.

Codon Adaptation Index (CAI) is a measure of synonymous codon usage bias and can be used to evaluate the extent of which codon usage of a supposed foreign gene is similar to highly expressed genes in the host genome [40]. CAI can range between 0 and 1 and values closer to 0 imply equal use of synonymous codons whereas values closer to 1 imply strong codon usage bias. MOQ_006101 displayed a CAI value of 0.518 (Figure 5C). In contrast, the median CAI across all genes was 0.545 ± 0.05 (median ± median absolute deviation). Thus, CAI was lower than the mean but still within the expected range, suggesting that the gene has conformed to the host genome.

Overall, these findings point to that MOQ_006101 was acquired by the lineage leading to *Tcm* rather than lost in *T*. *c*. *cruzi* and demonstrates an example of horizontal gene transfer between a photosynthesizing organism and a protozoan parasite. Interestingly, a genome comparison of two strains of the protozoan *Giardia intestinalis* also identified a strain-specific acetyltransferase [28]. Finally, the biological function of MOQ_006101, if any, remains to be determined.

### Comparison of synteny reveals putative rearrangements

*T*. *c*. *cruzi* has previously been reported to exhibit extensive DNA content and karyotype variability [24,41-43]. We investigated sequence co-linearity of the assembled data and compared with the current chromosome-level assembly of *Tcc* CLBR. Scaffolds >25 kb were extracted from the assemblies, which resulted in 307 and 229 scaffolds for *Tcm* and *Tcc* X10 respectively, amounting to 50.7% (19.6 Mb/38.6 Mb) and 44.7% (19.4 Mb/43.4 Mb) of the genomes. In order to identify putative inter- and intra-chromosome rearrangements, scaffolds were queried against *Tcc* CLBR (non-Esm) using the alignment program promer [44]. The number of chromosome hits per scaffold was plotted and the results were inspected. A total of 73 (*Tcm*) and 114 (*Tcc* X10) scaffolds contained hits to more than one chromosome from *Tcc* CLBR. However, manual examination showed that the vast majority of these hits were to gene family members (e. g. DGF, *trans*-sialidase, TcMUCII mucin) or other repeats. Hence, these were not likely to be rearrangements between chromosomes. 4 scaffolds were identified from *Tcm* (244, 732, 1101 and 2169) and 6 from *Tcc* X10 (94, 737, 1353, 2784, 2065 and 2359) that were involved in inter-chromosome rearrangements (Additional file 7: Figure S5). Moreover, *Tcm* and *Tcc* X10 both contained rearrangement in a region on chromosome 34, containing a repeat-like composition in *Tcc* CLBR. Scaffold 1101 from *Tcm* aligned with chromosomes 34 and 27. Scaffold 94 from *Tcc* X10 aligned with chromosomes 34 and 12. Also, scaffold 732 from *Tcm* aligned with the distal parts of *Tcc* CLBR chromosomes 22 and 42. In *Tcm*, VIPER elements were frequently found in regions where synteny was discontinued. Regions where rearrangements had occurred were frequently found inside unidirectional gene clusters.

Intra-chromosome rearrangements were searched for using the same strategy. This identified 23 and 13 scaffolds in *Tcm* and *Tcc* X10 respectively, where intra-chromosome rearrangements were identified. Frequently, one or several genes were found to have shifted location and were found to be located distally on the same chromosome. In a few cases, a certain structural variant was present in *Tcm* and *Tcc* X10 but not in *Tcc* CLBR, suggesting that it was introduced in the lineage leading to *Tcc* CLBR. *Tcm* scaffold 836 contained a large inverted region, flanked by VIPER elements. This inversion causes disruption of a head-to-head strand switch region. The larger number of structural rearrangements in *Tcm* likely reflects its phylogenetic distance from *T*. *c*. *cruzi*.

PCR validation was performed in order to validate the accuracy of the assembly and some of the identified rearrangements. Representative regions were selected from *Tcm*, *Tcc* X10 and *Tcc* CLBR and targeted for PCR amplification. The size of the PCR product was compared with the *in silico* expected size and confirm assembly consistency. In total, 3 of 4 genomic regions were successfully amplified from *Tcm*, 2 of 2 from *Tcc* X10 and 1 of 2 from *Tcc* CLBR (Additional file 8: Figure S6). Of which *Tcm* yielded the following PCR product sizes: 4, 0.5 and 0.8 kb. The first and second PCR products spanned across assembly gaps and therefore did not allow estimation of the expected sizes, but confirmed contigs to be accurately linked together. The third PCR reaction from *Tcm* resulted in the expected product size of 0.8 kb. *Tcc* X10 resulted in PCR products of sizes 0.8 kb and 1 kb, which were expected. The *Tcc* CLBR reaction resulted in the expected product size of 3 kb. Two PCR reactions did not work, which could either be due to non-optimal PCR conditions, formation of primer-secondary structures/duplexes or misassembly.

It is important to note that the present analyses are limited by the sequence continuity of the particular scaffold and therefore the presented numbers of observed rearrangements are likely to be underestimates. In conclusion, the majority of analyzed genomic regions from *Tcm* and *Tcc* X10 exhibited conserved local synteny with *Tcc* CLBR. However, insertions, deletions or other types of structural alterations occasionally interrupted synteny. These observations suggest that different *T*. *c*. *cruzi* lineages contain distinct karyotypes and other types of structural features, which have been fixed in a certain lineage. The cause of these rearrangements could either be due to random processes, i.e. oxidative stress or mistakes introduced by spontaneous cellular processes or perhaps less likely, physiological processes. Clearly, the presence of genetic variation other than SNPs provides an additional layer of complexity to studies of *T*. *c*. *cruzi* genetic variability.

### Widespread occurrence of copy number variation in *Tcm* and *Tcc* X10

**Figure 6:**
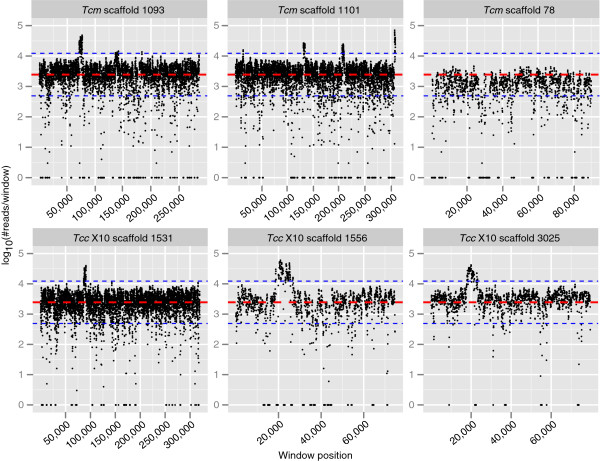
**Duplicated regions of *T***. ***c***. ***marinkellei *and *T***. ***c***. ***cruzi *Sylvio X10 inferred from short reads.** Short read coverage on assembly scaffolds of *T*. *c*. *marinkellei* B7 and *T*. *c*. *cruzi* Sylvio X10. Coverage was counted in 100 bp sliding windows along the scaffolds, with 50 bp step size. Each dot represents a 100 bp window. The horizontal axis shows the position of the window along the scaffold and the vertical axis shows the log_10_-scaled coverage of each window. Coverage was incremented by 1 to avoid infinite values for empty regions. Red lines show the global median coverage and blue lines show +/− 2X of the median absolute deviation. On scaffold 1093 (*Tcm*) the amplified region contained a dynein light chain protein, a nucleoside transporter and two genes of unknown function. On scaffold 1101 the first amplified region was 3 kb and contained a gene of unknown function, the second region was 3.7 kb and contained a pyruvate phosphate dikinase gene, the third region was 1.2 kb and contained a gene of unknown function. Scaffold 1531 (*Tcc* X10) contained a 4.7 kb amplification which contained a nucleoside transporter. Scaffold 1556 (*Tcc* X10) contained two genes of unknown function. Scaffold 3025 (*Tcc* X10) contained a 5 kb amplification with a copper-transporting ATPase gene. Scaffold 78 from *Tcm* showed evidence of aneuploidy, as the mean coverage was lower than for the other scaffolds.

Copy number variation has been reported from *T*. *c*. *cruzi* strains [33,34,45]. Such variation may represent important strain-specific characteristics, yet little is known about how *T*. *cruzi* lineages differ in this aspect. In the present study we investigated the occurrence of copy number variation in *Tcm* and *Tcc* X10 using short read depth (coverage). RT-qPCR was used to confirm some of the identified variations. Illumina reads were aligned to scaffolds >50 kb and a sliding window analysis was used to identify regions which exhibited higher than average coverage. Coverage was calculated in 100 bp windows with 50 bp overlap, i.e. the coverage of each position in the window was summed and log_10_-scaled. The baseline coverage was determined for each genome and was used to assess if a region displayed elevated coverage compared to the rest of the genome. The baseline was calculated as the median and median absolute deviation (mad) of log_10_-scaled coverage from all windows of one genome. This resulted in the baseline coverage (median ± mad) 3.39 ± 0.35 and 3.39 ± 0.33 for *Tcm* and *Tcc* X10 respectively. A duplicated region was defined as a stretch of 1,000 bp containing at least 5 windows above 2X the median standard deviation from the baseline. If two or more stretches were adjacent to each other, these were merged and counted as one region. This identified 142 and 182 duplicated regions in *Tcm* and *Tcc* X10. The duplicated region was not always restricted to one gene. On scaffold 1093 (*Tcm*), the amplified region was 6 kb and contained four coding sequences, including a nucleoside transporter and a dynein gene (Figure 6). The same region was also found amplified in *Tcc* X10 (scaffold 1531; Figure 6). Interestingly, a nucleoside transporter has been implicated in drug resistance in *Trypanosoma brucei*[46]. Housekeeping-genes were also found amplified, for example, paraflagellar rod protein 3 (MOQ_003131) from *Tcm* gave rise to a CNV signal. RT-qPCR with primers targeting this gene resulted in Ct=13.60, compared with Ct=15.3 for 8-oxoguanine DNA glycosylase (MOQ_000430), which lacked a CNV signal. Moreover, the prostaglandin F2 alpha synthase gene (MOQ_004364) gave rise to a CNV signal, and RT-qPCR resulted in Ct=12.41. In *Tcc* X10, one example of CNV is in the epsilon tubulin gene (TCSYLVIO_007352), for which RT-qPCR resulted in Ct=9.29. Surface antigens were frequently found amplified; a mucin-like gene on scaffold 1070 (*Tcm*), a surface protease GP63 on scaffold 1108 (*Tcm*). Scaffold 1109 (*Tcm*) contains an amplification of cystathionine beta-synthase, scaffold 1420 (*Tcm*) contains an amplification of NAD(P)-dependent steroid dehydrogenase, scaffold 143 (*Tcm*) contains an amplification of ferric reductase. There was also evidence of differential copy number variation, suggesting amplification in one genome but not the other. One example is the amplification of a pyruvate phosphate dikinase on scaffold 1101 in *Tcm*, which does not give rise to a CNV signal in *Tcc* X10.

In order to identify chromosomal aneuploidies, we calculated the baseline coverage for each scaffold. Scaffolds with a median <3.2 and median absolute deviation <0.7 were extracted (empirically determined thresholds). This identified 7 scaffolds in *Tcm* with a lower average coverage: 950, 938, 79, 78, 70, 2392 and 2744. These genomic regions were homologous with chromosomes 25, 25, 12, 12, 12 and 7 in *Tcc* CLBR. No scaffolds with low overall coverage were identified in *Tcc* X10. This suggested the existence of monosomic chromosomes in *Tcm* but not in *Tcc* X10. As expected, heterozygosity was absent in these putatively monosomic *Tcm* regions. However, the homologous region in *Tcc* X10 displayed heterozygosity. This further supported the likely monosomic state of these regions in *Tcm*. Genomic qPCR with a primer pair targeting scaffold 78 in *Tcm* resulted in Ct=17.81 for the putative monosomic scaffold, whereas for putative disomic regions on scaffold 1093 and 1101 Ct was 15.08 and 15.30. Moreover, we searched for evidence of higher ploidy levels. Scaffolds with median > 3.5 were extracted. This identified 14 and 5 scaffolds in *Tcm* and *Tcc* X10 respectively, with an increased overall coverage. These scaffolds showed homology with large mega-base chromosomes from *Tcc* CLBR, suggesting that higher ploidy levels may be more common in larger chromosomes.

The presented analysis confirms that copy number variation is a common feature of the *Tcm* and *Tcc* X10 genomes. In theory, copy number variation would not be beneficial for the parasite as it increases the amount of DNA that needs to be replicated and the energy cost of the cell. The evolutionary benefit of having such an excessive amount of genes would seem to be limited. It is possible that copy variation does not infer any evolutionary advantage for the parasite, but is only a consequence of sloppy or non-perfect DNA replication mechanisms of these parasites.

### Retrotransposons, repetitive elements and low complexity repeats

Transposons are present in most eukaryotes and contribute to genome size and plasticity [47]. Trypanosomatid genomes contain several families of dead and presumably active retrotransposons [48]. *Tcm* and *Tcc* X10 were searched for 11 classes of repetitive elements, including retrotransposons. 6.5% (2,344,982 Mb/34,233,090 Mb) and 9.9% (3,852,782 Mb/38,598,156 Mb) of the assembled bases corresponded to repetitive elements in *Tcm* and *Tcc* X10 respectively (Table 5). 8 of 11 repeat categories were more abundant in *Tcc* X10, with a total expansion factor of 1.26 in this genome compared with *Tcm* (8.2%/6.5%, Table 5).

**Table 5:** Comparison of repetitive elements

	***T. c. marinkellei***		***T. c. cruzi *Sylvio X10**		
**Element**	**# bp**^** a**^	**% Short reads**^** b**^	**# bp**^** a**^	**% Short reads**^** b**^	**SE**^** c**^
**VIPER**	574,697 (1.679 %)	1.535	1,116,378 (2.892 %)	1.811	*Tcc* X10
**DIRE**	433,619 (1.267 %)	1.156	655,064 (1.697 %)	0.907	*Tcm*
**L1Tc**	432,474 (1.263 %)	1.168	805,885 (2.088 %)	2.158	*Tcc* X10
**TcTREZO**	382,416 (1.117 %)	1.024	481,685 (1.248 %)	1.081	*Tcc* X10
**E22**	223,679 (0.653 %)	0.630	281,491 (0.729 %)	0.590	*Tcc* X10
**SIRE**	176,724 (0.516 %)	0.497	238,914 (0.619 %)	0.527	*Tcc* X10
**SZ23**	94,765 (0.277 %)	0.224	151,879 (0.393 %)	0.275	*Tcc* X10
**CZAR**	18,338 (0.054 %)	0.104	102,810 (0.266 %)	0.203	*Tcc* X10
**NARTc**	4,705 (0.014 %)	0.010	10,936 (0.028 %)	0.020	*Tcm*
**C6**	2,944 (0.009 %)	0.006	167 (0.000 %)	0.000	*Tcm*
**TCSAT1**	621 (0.002 %)	0.149	7,573 (0.020 %)	0.628	*Tcc* X10
**Total**	**2**,**344**,**982** (**6**.**851**%)	**6**.**503**%	**3**,**852**,**782** (**9**.**98**%)	**8**.**200**%	

^a^ The sum of masked base pairs in the assembly. The number inside parenthesis refers to the percentage of assembled bases.

^b^ The percentage of short reads that was mapped on these features.

^c^ SE=Significantly Enriched. Refers to if one genome contained significantly more of this gene family. The significance was determined from an empirical distribution of read depth differences from homologous regions of *Tcm* and *Tcc* X10, corrected for genome size. The empirical distribution was used to calculate a *p*-value.

The Long Terminal Repeat (LTR)-like retroelement VIPER [49] belongs to the superfamily tyrosine recombinase retrotransposons [50] and was the most abundant element in *Tcm* and *Tcc* X10 respectively, representing 24.5% (574,697 Mb/2,344,982 Mb) and 28.9% (1,116,378 Mb/3,852,782 Mb) of the repetitive elements (Table 5). The large amount of sequence related to these elements suggested that large-scale proliferation occurred before the split of *T*. *c*. *cruzi* and *T*. *c*. *marinkellei*. Furthermore, phylogenetic reconstruction based on a multiple sequence alignment (MSA) indicated some substructure between *Tcc* X10 and *Tcc* CLBR, whereas *Tcm* in large formed a more distant clade (Additional file 9: Figure S7). 69% (3,450/4,968) of the MSA sites were too diverged to be included in the multiple-alignment, suggesting that these elements have been inactive for a substantial time. The human infecting lineage does contain a larger amount of these elements, possibly due to a loss of VIPER-related sequences in *Tcm*. In conclusion, repetitive elements explain in part the smaller genome size of *Tcm*. 3 repeat categories were on the contrary expanded in *Tcm*; the 2 low-abundance repeats NARTc and C6 and the abundant DIRE (degenerate Ingi/L1Tc-related retroelement) element.

*De novo* repeat discovery was performed in order to identify unique sequence repeats, using the program RepeatScout [51] and RepeatMasker [52]. RepeatScout identified 2,225 (*Tcm*) and 2,263 (*Tcc* X10) repeats of variable lengths. These repeats were then filtered using these criteria; i) removal of repeats shorter than 50 bp, ii) removal of repeats containing more than 50% low complexity sequence, iii) removal of repeats with fewer than 10 genomic copies, iv) removal of known repeats (i.e. present in *Tcc* CLBR). This decreased the number of hits to 20 (*Tcm*) and 3 (*Tcc* X10) using the outlined criteria. Manual examination of the *Tcm* repeats revealed that 12 corresponded to diverged *Tcc* CLBR sequences, including a spliced leader sequence and sequences related to MASP and TcMUCII mucin. We searched for these 8 repeats in the genome assemblies of *Tcc* X10 and *Tcc* CLBR as well as in raw reads, which decreased the number of *Tcm*-specific repeats to 7. The length of the identified *Tcm*-specific repeats varied between 60 to 896 bp, and BLAST searches resulted in non-significant hits to sequences of metazoan origin. These repeats were found exclusively on short contigs (0.5-1 kb), corroborating the idea that the repetitive components of these genomes have evolved faster. We estimated the copy number of the two longest repeats, Tcm-Rep1 (825 bp) and Tcm-Rep2 (896 bp) from the depth of 454 read coverage mapped on these sequences. The average 454 read coverage (12x) was then used to estimate copy number. The average read coverage was 1974 reads/position for Tcm-Rep1 and 1,494 reads/position for Tcm-Rep2. Hence, the estimated copy number became 164 and 124 for Tcm-Rep1 and Tcm-Rep2 respectively. Taken together, these two repetitive elements contribute ~250 kb of sequences to the *Tcm* genome and also represent a large set of putative *Tcm*-specific sequences. Since the repeats were not found in *Tcc* X10 and *Tcc* CLBR, it is possible that a loss has occurred in the lineage leading to the human infective *T*. *c*. *cruzi*.

### *T*. *c*. *marinkellei* invades non-bat epithelial cells in small numbers and divides intracellularly

**Figure 7:**
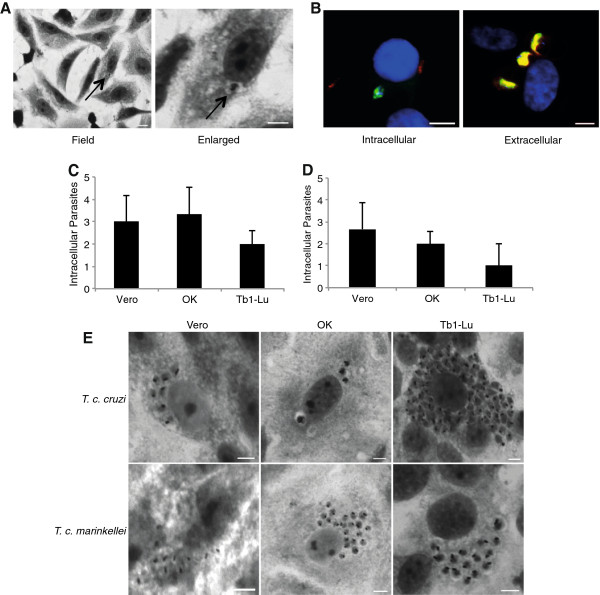
**Cell invasion assay.** (**A**) Intracellular *T*. *c*. *marinkellei* parasites stained with Giemsa. Scale bars correspond to: 10 μm (field) and 5 μm (enlarged). (**B**) Immunolabelled intracellular and extracellular *T*. *c*. *marinkellei* parasites. Intracellular parasites were labelled with anti-WCB antibody (green), while extracellular parasites were labelled with anti-WCB antibody (green) and *anti*-*T*. *c*. *marinkellei* serum (red), which superimposed gives the yellow color. Nuclei and kinetoplasts were counter stained in DAPI (blue). (**C**) Number of intracellular *T*. *c*. *marinkellei* parasites in the Giemsa assay. (**D**) Number of intracellular *T*. *c*. *marinkellei* parasites in the immunolabeling assay. (**E**) Intracellular *T*. *c*. *cruzi* and *T*. *c*. *marinkellei* parasites in three different cell types. *T*. *c*. *cruzi* and *T*. *c*. *marinkellei* parasites were incubated for 5 days with Vero (monkey), OK (opossum) and Tb1-Lu cells before Giemsa staining. Two hundred cells were assayed in 3 independent experiments for Giemsa and immunolabeling assays. The scale bars correspond to 5 μm.

Experimental infections were performed on three mammalian cell lines to further understand the potential of *Tcm* to invade non-bat derived cells. The following lines of epithelial cells were used; Vero cells (kidney cells from African green monkey), OK cells (from a North American opossum) and Tb1-lu cells (bat lung). *Tcm* metacyclic trypomastigotes were incubated overnight with cells from each cell line (Materials and Methods). Extra- and intracellular parasites were immunolabelled using *Tcm* and *Tcc* positive sera and anti-whole cell body antibody (Figure 7). In parallel, intracellular parasites were stained with Giemsa dye. Both experiments independently showed that *Tcm* is capable of invading each of the three cell lines. *Tcm* did not show a particular preference for the bat cell line.

We also investigated the ability of *Tcm* to replicate intracellularly using the same cell lines. The human infective *Tcc* was included as a positive control. Infected cells were incubated over a 5 day time course, the development of intracellular amastigotes during this period indicated that *Tcm* is capable of intracellular proliferation (Figure 7). Amastigogenesis and amastigote proliferation of *Tcm* following metacyclic invasion appeared to be analogous to *Tcc* controls. These data suggested that *Tcm* is capable of infecting other cells than strictly those from bats, and hence, that the infection is not blocked by species-specific host cell tropism mediated at the level parasite entry. In conclusion, the infection barrier must therefore arise in a different context, though whether this relates to different aspects of *Tcm* specific biology or as physiological or immunological differences between hosts, or as a combination of both, remains to be elucidated.

## Conclusions

This study is the first genome analysis of a non-human associated member of *Schizotrypanum*. Our aim was to identify genome sequence differences that may relate to host specificity or other phenotypical differences, as well as to further understand the evolution of these parasite lineages. We found a slightly smaller genome of *T*. *c*. *marinkellei* compared with the human infective strains, although it remains an open question if this is a general tendency among bat-associated trypanosomes. *T*. *c*. *marinkellei* and *T*. *c*. *cruzi* shared the same set of core genes, i.e. there were no missing coding sequences in terms of housekeeping genes. On the contrary, several gene families were expanded in *T*. *c*. *cruzi* Sylvio X10, contributing to the larger genome size. This suggested that *T*. *c*. *cruzi* Sylvio X10 have a more versatile toolbox of surface antigens, which may reflect an adaptation to its host. Interestingly, one subspecies specific acetyltransferase gene was identified in *T*. *c*. *marinkellei*, containing detectable homology with genes from photosynthesizing organisms. It appears likely that this gene was acquired after the split of *T*. *c*. *cruzi* and *T*. *c*. *marinkellei*, since the gene was missing from *T*. *c*. *cruzi* strains Sylvio X10 and CL Brener. The gene represents a rare example of gene transfer between distantly related eukaryotes and may provide additional functionality to *T*. *c*. *marinkellei*. Future efforts will be required to understand its function. Considering the divergence time between *T*. *c*. *marinkellei* and *T*. *c*. *cruzi* (~6.5-8.5 MYA [10-12]), remarkably few absolute gene differences were present. This suggests that the core gene content of *T*. *cruzi* lineages is relatively stable, whereas the repetitive component is allowed to undergo more rapid changes. The low number of subspecies specific genes suggests that phenotypic variation, like host specificity, might be encoded by more discrete variation, e.g. via non-synonymous nucleotide variants leading to specific protein isoforms. The difficulty to explain how the genome encodes phenotypes like host-specificity is further illustrated by our finding that *T*. *c*. *marinkellei* invades non-bat cells, which indicates that the machinery to invade host cells is functionally conserved. The two subspecies *T*. *c*. *marinkellei* and *T*. *c*. *cruzi* were on average ~7.5% diverged in coding sequences with respect to single nucleotide differences. The large number of small nucleotide differences may have implications on phenotypic variation via the formation of new alleles. The present study has provided many new candidate genes, including putative antigens that can provide starting points for functional investigation of phenotypic variation of these parasite lineages.

Extensive copy number variation of various genes was identified. Copy number variation has been suggested as means for the parasite to increase gene expression in the absence of transcriptional regulation. These findings are not surprising and are corroborated by the long-standing knowledge of genomic variability in *T*. *c*. *cruzi*[24,33,41-43]. It is possible that phenotypes may be encoded at the transcriptional level. Interestingly, the *T*. *c*. *marinkellei* and *T*. *c*. *cruzi* genomes also contained variation in the amounts of non-coding repeats, related to retroelements and other previously uncharacterized repeats. As these differences were substantial, it remains plausible that whole chromosomes or chromosomal chunks have been lost in *T*. *c*. *marinkellei*. None of the larger chromosomes were missing, suggesting that smaller chromosomes harboring surface antigens or other repeats have been lost. The plasticity of the *T*. *cruzi* karyotype is further demonstrated by the fact that certain chromosomes appear to be monosomic in one subspecies but not in the other. The existence of such monosomic chromosomes reduces allelic redundancy and might have implications on transcript abundance. Karyotype variability therefore stands as another possible source of phenotypic variation. Finally, the amount of intraspecific genetic variation identified in this study is likely to represent only the tip of the iceberg in terms of the actual genetic variation present in natural reservoirs.

Taken together, the presented draft genomes raise further questions about genome evolution and diversity in this group of protozoa, and the putative functional implications of this variation. Further exploration of the genetic diversity within *Schizotrypanum* should therefore be a future priority as this may help to resolve complex relationships between parasites, vectors and hosts. The decreasing cost and time for whole genome sequencing should therefore pave the way for further large-scale efforts to understand the underlying genetic basis of these parasites.

## Methods

### Accession numbers

Sequence data and annotations have been deposited in NCBI GenBank under the accession numbers AHKC01000000 (*T*. *c*. *marinkellei* B7) and ADWP02000000 (*T*. *c*. *cruzi* Sylvio X10). The data can also be downloaded from http://www.ki.se/chagasepinet/genomes.html.

### Cell culture, library preparation and sequencing

*T*. *c*. *marinkellei* B7 clone 11 and *T*. *c*. *cruzi* Sylvio X10 clone 1 were cultured using standard conditions (supplemented RPMI + 10% FBS). Parasite DNA was extracted using the Puregene kit. Genome size estimation of *T*. *c*. *marinkellei* was performed using flow cytometry as described by Lewis *et al*. [24]. Illumina sequencing: The *Tcm* and *Tcc* X10 mate pair libraries were prepared according to Van Nieuwerburgh *et al*. [22]. Initially, the paired-end protocol for 3 kb libraries from Roche/454 was used until circularized. After circularization, the libraries were prepared following the Illumina mate-pair protocol: 5 μg of genomic DNA was sheared to approximately 3 kb and end-polished. Fragments were then ligated to the Roche/454 circularization adapters and size selected using AMPure beads (Beckman Coulter). The ligated DNA was circularized using Cre-recombinase and then exonuclease treated. All enzymes were obtained from New England Biolabs. The circular DNA was fragmented using Covaris S2, end-repaired and purified using streptavidin coated magnetic beads. The DNA was then A-tailed and ligated with sequencing adapters and PCR amplified. The post-circularization steps were performed using the reagents either supplied or referenced by the Illumina mate-pair protocol. The clustering was performed on a cBot cluster generation system using a HiSeq paired-end read cluster generation kit. The samples were sequenced on an Illumina HiSeq2000 as 2x100-nt reads (one lane for each genome). Base conversion was done using Illumina OLB v1.9. 454 sequencing: Prepared according to the manufacturer’s instructions and sequenced on a 454 instrument with Titanium chemistry.

### Sequence assembly

Illumina reads were quality filtered and trimmed using the fastq_quality_filter program of the fastx toolkit (parameters: -*q 20* -*p 95*). Illumina reads were assembled with Velvet v1.1.04 [53], using the empirically determined kmer length of 43 and a minimum contig length of 500 bp. *velveth* and *velvetg* were called with the following commands, respectively: ‘<name> 43 -fastq -shortPaired1 input.fastq’ and ‘<name> −min_contig_lgth 500 -exp_cov auto -ins_length 2000 -ins_length_sd 2000 -amos_file no -scaffolding no -unused_reads yes’. Unused reads were extracted and subjected to a second round of Velvet assembly using a kmer length of 53 (empirically determined) and a minimum contig length of 400 bp (velvetg: ‘-min_contig_lgth 400 -exp_cov auto -ins_length 10 -ins_length_sd 5000 -scaffolding no’). 454 reads were assembled with CELERA v6.1 [54] (default settings). 454-related insertion-deletion errors in the assembly were corrected using the Illumina reads: Illumina reads were aligned with bwa [55], and the resulting SAM file was then scanned in order to identify indels. In order to correct a position, at least 10 Illumina reads were required to support the change. The three assemblies (i. Illumina Velvet 1; ii. Illumina Velvet 2; iii. 454 CELERA) were pooled and merged into a non-redundant assembly. Assembly merging was performed using the Zorro pipeline [56], relying on minimus2 and mummer to split and merge contigs. The merged assembly was filtered to include only contigs longer than 500 bp. Maxicircle (mitochondrial) sequences were identified using megablast and removed. Minicircle (mitochondrial) sequences were identified and removed by searching for the TCKIN2 signature sequence. Distance information from mate-pairs were used to order and orient contigs into scaffolds. The software SSPACE was used for scaffolding [57]. A small number of intra-scaffold gaps (~200/genome) could be closed using the overlap between adjacent contigs. The final assembly was subjected to gap closure using the IMAGE pipeline [23].

### Bioinformatics analyses

Annotation: Gene models were transferred from *Tcc* CLBR using Perl scripts, and additional genes were called using GeneMarkS [58]. Annotations were manually curated using the Artemis Comparison Tool [38]. Orthologous genes were identified using the best reciprocal BLASTp hit (E-value 1e-10). Unique genes were identified using BLASTp and tBLASTn searches. Genes in synteny were determined using homology of surrounding genes. At least one adjacent homologous gene was required to be present in order to call a gene syntenic. dN and dS values [59] were calculated using the yn00 program of the PAML package [60]. Rearrangements: Chromosomal re-arrangements were identified from alignments generated using promer [44]. Repeats: Sequence repeats were identified with RepeatMasker [52], Repbase [61] and Tandem Repeat Finder [62]. Phylogenetic analysis of the candidate horizontal gene transfer: A dataset was extracted with Blast Explorer [63] (E-value <1e-40). Multiple sequence alignment was done with ClustalW v2.1 [64] and filtered with Gblocks [65] to remove ambiguous positions. Alignments were manually inspected in Jalview [66]. A maximum likelihood tree was generated with RAxML v7.0.4 [67], using the PROTGAMMAJTT model and 100 bootstrap replicates. Multicopy genes: Genome sequence reads (Illumina) were mapped back to the assembly using bwa (default settings) [55] and the coverage was calculated. Sequence entropy was calculated using bio3d [68]. Copy number variation: Genome sequence reads (Illumina) were mapped back to the assembly and the reference sequence was divided into 100 bp windows with 50 bp overlap. The sum of coverage for each position in the window was computed, log_10_-scaled and plotted. Heterozygosity: Reads were aligned with the assembly and samtools [69] and awk were used to extract polymorphic positions. Maxicircle analysis: Manual annotation of maxicircle coding regions was performed by comparison to the published CLBR (GenBank: DQ343645), Esmeraldo (GenBank: DQ343646) and Sylvio X10/1 (GenBank: FJ203996) maxicircle coding sequences. Sequence identity was calculated using BioEdit v7.0.9.0 [70]. Heteroplasmy was called with samtools mpileup [69]. A SNP was defined as a nucleotide variant present in at least 5 independent reads (with parameters: 20X coverage and mapping quality, 30). All scripts are available from the authors on request.

### Normal PCR and quantitative real-time PCR

Normal PCRs: Primers were selected with Primer3 [71] and synthesized by Sigma-aldrich. Amplification was performed using the Phire Hot Start II DNA polymerase kit (Finnzymes). The targets were amplified in a mixture containing 1X Phire Reaction Buffer, 0.2 mM of dNTPs (Fermentas), 0.4 μM of each primer, 2% DMSO, 50 ng of genomic DNA, 0.4 μl of Phire Hot Start II DNA polymerase and water to a final volume of 20 μl. The cycling conditions were as follows: Initial denaturation at 98°C for 2 minutes, 35 cycles of 98°C for 10 seconds, 60–68°C for 10 seconds and 72°C for 10 seconds and a final extension step at 72°C for 2 minutes. The Tc_CLB1 amplicon of 3 kb size uses a 45 seconds extension step, in comparison with the other amplicons that uses just 10 seconds. Amplicons were visualized using a 1.3% agarose gel stained with ethidium bromide. Quantitative Real-time PCR (RT-qPCR) on the specific acetyltransferase gene in *Tcm*: RNA extraction was performed using the RNeasy Mini kit (Qiagen). RNA was converted to cDNA with reverse transcriptase and random hexamer primers. Reactions were performed using Power SYBR Green MasterMix (Life Technologies) under standard conditions. Template concentration was 50 ng/μl and 1 μl of template was used in each reaction. Primer concentration was 0.2 μM in 20 μl of final volume. Each experiment was performed in triplicate and the average cycle threshold (Ct) value was used as a measurement of initial template abundance. All reactions were performed on an ABI 7300 Real-time PCR system. The following primer pairs were selected for the experiment (5^′^ to 3^′^); unique gene: TTGCAGCATATGTGTGGATG (F), ACGTTAAAGAAACGGCTGCT (R), hypoxanthine-guanine phosphoribosyltransferase: GCCTTCATGTCAACCCTCTT (F), AAGACGTGACACCTTCACCA (R), 18S rRNA: TTACGTCCCTGCCATTTGTA (F), TTCGGTCAAGTGAAGCACTC (R). RT-qPCR to validate copy number variation: Experimental conditions were similar as for the previous experiment, except that genomic DNA was used (20 ng/μl, 1 μl loaded).

### Cell invasion assay

Vero cells were maintained in DMEM + glutaMAX (Gibco, Invitrogen, UK) supplemented with 10% fetal calf serum (PAA laboratories, UK), 5mM l-glutamine, 50 μg/ml streptomycin and 50 units/ml penicillin. Tb1 lu cells (HPACC, UK) were maintained in MEM (ATCC, UK) and supplemented as described above. OK cells (HPACC, UK) were maintained in MEM (Sigma-Aldrich, UK) with 10% fetal calf serum, 5 mM l-glutamine, 50 μg/ml streptomycin and 50 units/ml penicillin and 5% non-essential amino acids. *T*. *c*. *marinkellei* epimastigotes from lineage B7 cl11 were grown in Liver Infusion Tryptose (LIT) and *T*. *c*. *cruzi* strain M6241 was grown in RPMI for 2 weeks prior to experiments. Each cell line was seeded at a density of 10^5^ per ml onto 13 mm diameter coverslips and allowed to grow overnight. Cells were then washed and the growth media replaced with media containing 10^5^ metacyclic *T*. *c*. *marinkellei* and incubated at 37°C for either overnight or 5 days. The media was removed and cells were washed twice with PBS and either fixed with 4% paraformaldehyde for 20 min or ice cold methanol. Methanol fixed cells were stained with Giemsa for 10 min and imaged with a Zeiss Axioplan 2 microscope and a Zeiss AxioCam Hrc camera. Paraformaldehyde fixed cells were blocked in 10% goat serum and labelled with *T*. *c*. *marinkellei* positive serum for 1 h before incubating with AffiniPure Fab fragments (Stratech Scientific Ltd., UK) for 10 min, these epitopes were then recognised by anti-rabbit Alexa Fluor 568. The cells were blocked again in 10% goat serum and permeabilised in 1% NP40 for 3 min before labelling with anti-whole cell body (WCB) antibody [72] (kindly provided by Prof. K. Gull) for 1 h recognised by anti-mouse Alexa Fluor 488 and finally DAPI stained before mounting in Fluoromount (Sigma-Aldrich, UK). Antibody labelled cells were visualised by a Zeiss Axioplan 2 microscope and Zeiss AxioCam MRm camera all image processing was done with Axiovision 4.7 software. Two hundred cells were assayed in the overnight experiments and the results are expressed as an average of three independent experiments.

## Competing interests

The authors declare that they have no competing interests.

## Authors’ contributions

OF carried out the bioinformatics analyses and drafted the manuscript. CTL, SO, CEB, LAM, MDL, MSL carried out cell culture, flow cytometry, PCR experiments, cell invasion assays and participated in the bioinformatics analyses. BA, MAM, KMT, CJM conceived the study, and participated in its design and coordination and helped to draft the manuscript. All authors read and approved the final manuscript.

## Supplementary Material

Additional file 1 **Figure S1.** Flow cytometry analysis of the *T. c*. *marinkellei* genome size. Description: Fluorescence emission histograms for propidium iodide-labelled epimastigotes showing relative DNA contents of *T*. *c*. *cruzi* Esm/3 (TcII), *T*. *c*. *cruzi* Sylvio X10/4 (TcI) and *T*. *c*. *marinkellei* B7/11. Click here for file

Additional file 2 **Figure S2.** Histogram and smoothed density estimate of assembly-wide coverage differences between *Tcm* and *Tcc* X10. Description: (A) Histogram of percentage short read coverage differences from homologous regions. Percentages have been corrected for genome size. Vertical red lines indicate the lower and upper 2.5% quantiles. (B) Smoothed kernel density estimate of the left histogram created using logspline R package. Click here for file

Additional file 3 **Figure S3.** Sequence variation of the TcMUCII mucin gene family. Description: Entropy plots of the TcMUCII mucin gene family. TcMUCII mucin genes were extracted from *Tcm*, *Tcc* X10 and *Tcc* CLBR non-Esm. Sequences were aligned with ClustalW v2.1. Sequence entropy was calculated using the entropy function of the R package bio3d. Only alignment positions with less than 10% gaps were included in the analysis. The normalized entropy score was then plotted as a function of alignment position, where conserved sites (low entropy) score 1 and diverse (high entropy) sites score 0. The analysis indicated that 5′ and 3′ termini of TcMUCII mucin genes generally are the most conserved in all three genomes and that the central region is the most variable. Click here for file

Additional file 4 **Table S1.** Maxicircle gene coordinates and metrics. Description: Gene metrics for *T*. *c*. *cruzi* and *T*. *c*. *marinkellei* maxicircles. Including coordinates, average identity and length. Click here for file

Additional file 5 **Figure S4.** Maxicircle phylogenetic tree. Description: Maximum likelihood phylogenetic tree of the maxicircle sequences from *T*. *c*. *marinkellei*, *T*. *c*. *cruzi* Sylvio X10, *T*. *c*. *cruzi* CL Brener, *T*. *c*. *cruzi* Esmeraldo using *T*. *brucei* and *L*. *tarentolae* as outgroups. The full maxicircle sequences were aligned with ClustalW v2.1 and the subsequent alignment was filtered using Gblocks (default settings). The tree was inferred using MEGA v5.1 from 13,731 (49%) alignment positions. Click here for file

Additional file 6 **Table S2.** Ratio of non-synonymous and synonymous nucleotide substitutions. Description: Orthologous gene pairs between *T*. *c*. *marinkellei* and *T*. *c*. *cruzi* CL Brener displaying elevated dN/dS (> 1.1). The yn00 program was used to calculate dN and dS. Click here for file

Additional file 7 **Figure S5.** Disruption of sequence co-linearity. Description: Disruption of chromosomal co-linearity between *T*. *c*. *marinkellei* and *T*. *c*. *cruzi* CL Brener non-Esmeraldo-like (A) as well as between *T*. *c*. *cruzi* Sylvio X10 and *T*. *c*. *cruzi* CL Brener non-Esmeraldo-like (B). Black chromosomes prefixed with ‘Chr’ represent sequences from *Tc* CL Brener whereas white chromosomes prefixed ‘contig’ represent sequences from *Tcm* and *Tcc* X10 assemblies. Alignments were generated using the promer software (Kurtz *et al*., 2004). Chromosomal stretches marked with green color represent gaps in the assembly. Only gaps larger than 5 kb are shown. The most outer numbers are sequence identifiers. Click here for file

Additional file 8 **Figure S6.** PCR validation of synteny breaks. Description: PCR validation results from a few regions containing synteny breaks in *T*. *c*. *marinkellei* and *T*. *c*. *cruzi* Sylvio X10. Click here for file

Additional file 9 **Figure S7.** Phylogenetic tree of VIPER elements. Description: Maximum likelihood phylogenetic tree of VIPER retroelements from *T*. *c*. *marinkellei*, *T*. *c*. *cruzi* CLBR, *T*. *c*. *cruzi* X10. The colors correspond to; blue (*Tcm*), green (*Tcc* CLBR), red (*Tcc* X10). VIPER elements were identified with RepeatMasker and only elements longer than 2000 bp were included: 209 sequences in total (35 from *Tcm*, 57 from *Tcc* X10 and 117 from *Tcc* CLBR). The average branch lengths were; 0.0682 (*Tcm*), 0.039 (*Tcc* X10), 0.0455 (*Tcc* CLBR). The alignment was constructed with ClustalW and manually inspected. Gblocks was used to remove ambiguities from the alignment, which resulted in a total of 1518 positions that were used for inferring the phylogeny. The maximum likelihood tree was inferred with RAxML using the GTRCAT model and 100 bootstrap replicates. Click here for file
