# Supplementary figures and images for: Comparative genomic analysis of human infective Trypanosoma cruzi lineages with the bat-restricted subspecies T. cruzi marinkellei
Source: BMC Genomics. 2012 Oct 5;13:531. doi: 10.1186/1471-2164-13-531 (PMC3507753; doi:10.1186/1471-2164-13-531)

# Entropy plots for the TcMUCII mucin gene family

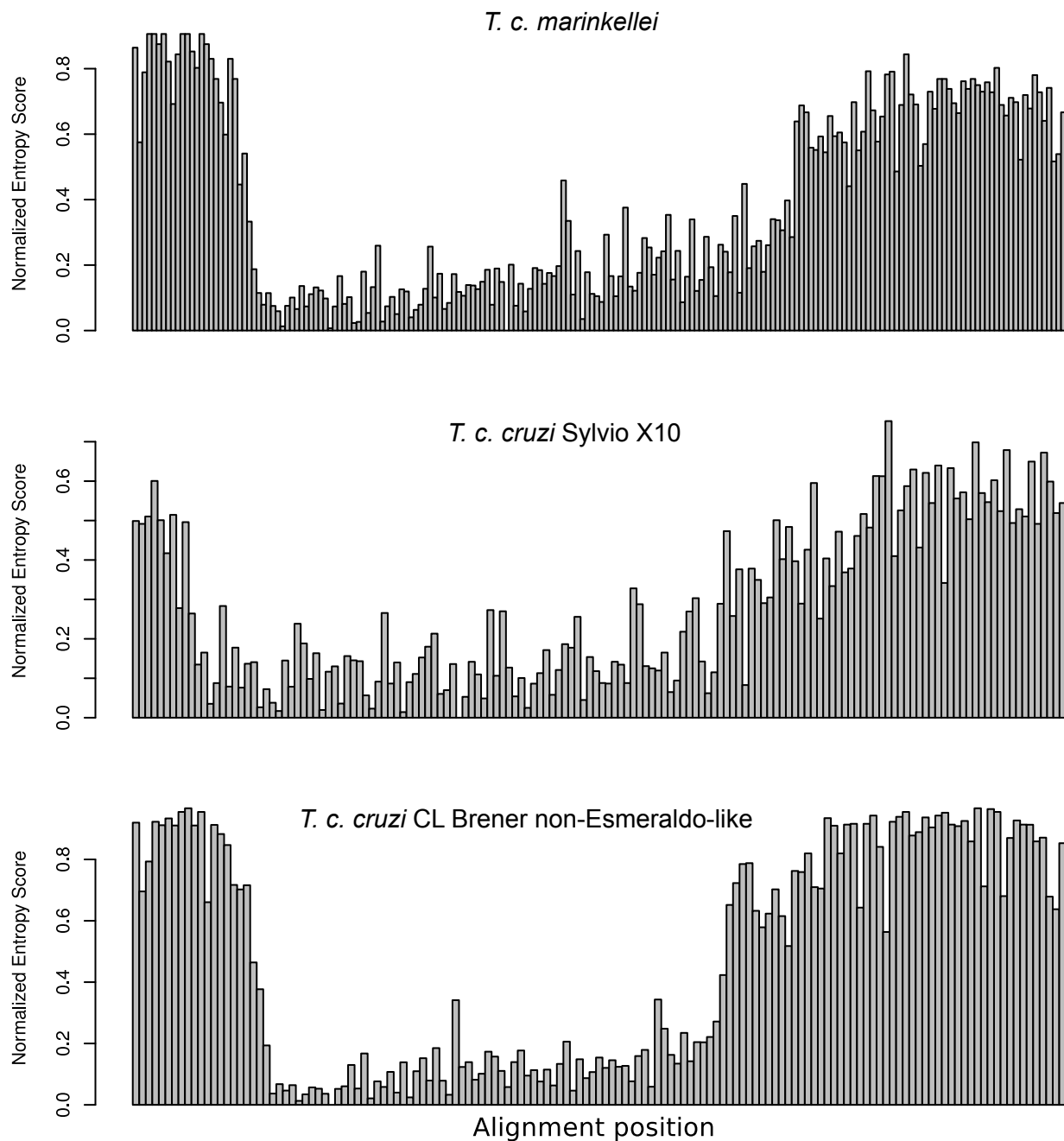

Supplement: Additional file 1 — Figure S1. Flow cytometry analysis of the T. c. marinkellei genome size. Description: Fluorescence emission histograms for propidium iodide-labelled epimastigotes showing relative DNA contents of T. c. cruzi Esm/3 (TcII), T. c. cruzi Sylvio X10/4 (TcI) and T. c. marinkellei B7/11. [file 1471-2164-13-531-S1.pdf]

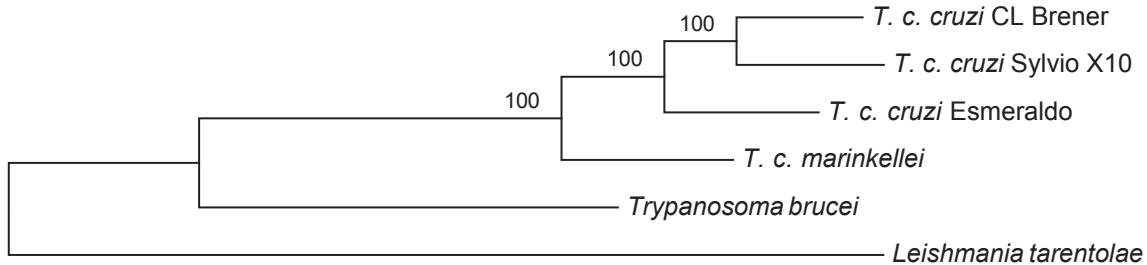

0.05

Supplement: Additional file 2 — Figure S2. Histogram and smoothed density estimate of assembly-wide coverage differences between Tcm and Tcc X10. Description: (A) Histogram of percentage short read coverage differences from homologous regions. Percentages have been corrected for genome size. Vertical red lines indicate the lower and upper 2.5% quantiles. (B) Smoothed kernel density estimate of the left histogram created using logspline R package. [file 1471-2164-13-531-S2.pdf]

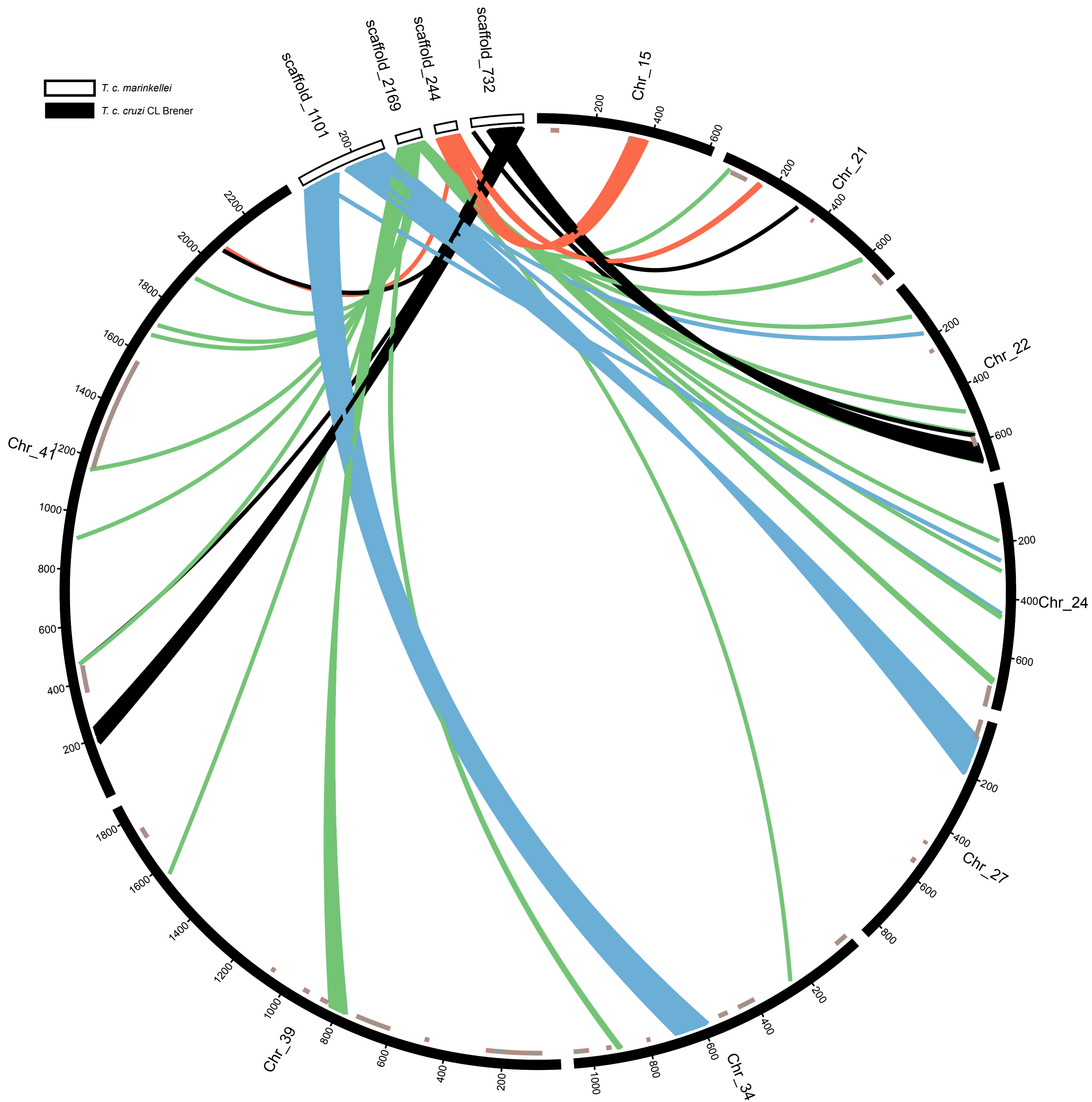

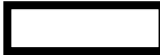 *T. c. cruzi* Sylvio X10  
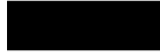 *T. c. cruzi* CL Brener

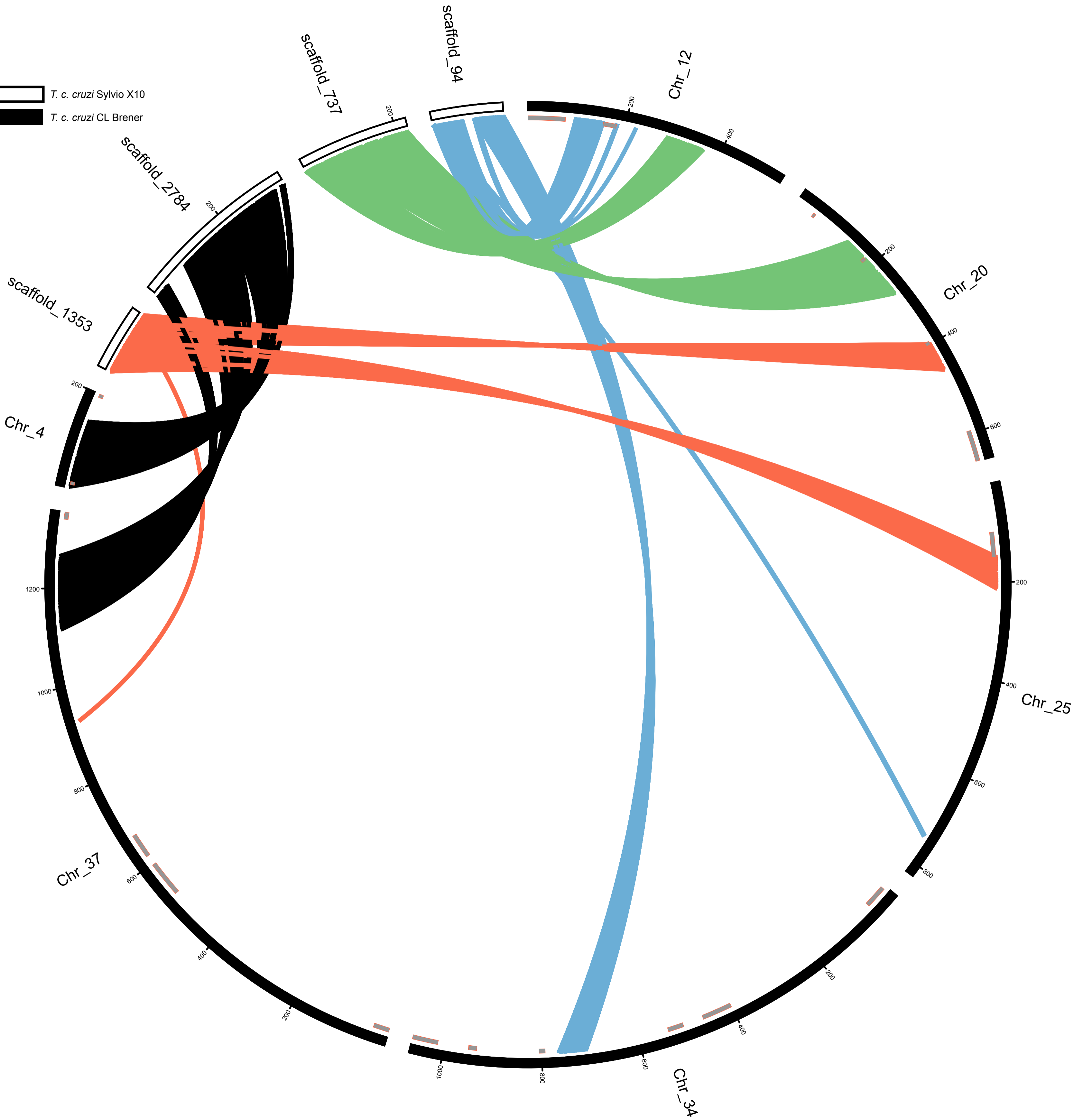

Supplement: Additional file 3 — Figure S3. Sequence variation of the TcMUCII mucin gene family. Description: Entropy plots of the TcMUCII mucin gene family. TcMUCII mucin genes were extracted from Tcm, Tcc X10 and Tcc CLBR non-Esm. Sequences were aligned with ClustalW v2.1. Sequence entropy was calculated using the entropy function of the R package bio3d. Only alignment positions with less than 10% gaps were included in the analysis. The normalized entropy score was then plotted as a function of alignment position, where conserved sites (low entropy) score 1 and diverse (high entropy) sites score 0. The analysis indicated that 5′ and 3′ termini of TcMUCII mucin genes generally are the most conserved in all three genomes and that the central region is the most variable. [file 1471-2164-13-531-S3.pdf]

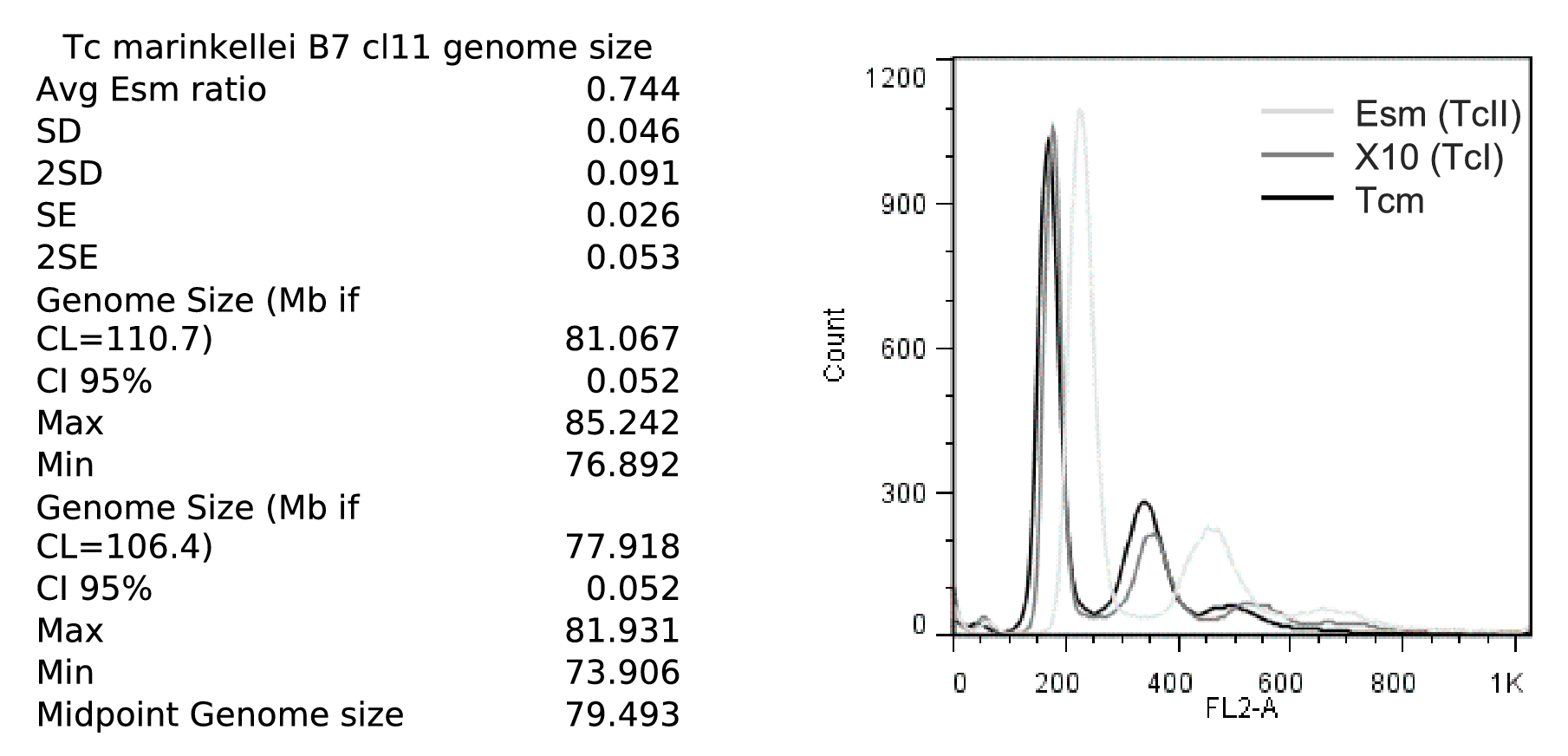

Supplement: Additional file 4 — Table S1. Maxicircle gene coordinates and metrics. Description: Gene metrics for T. c. cruzi and T. c. marinkellei maxicircles. Including coordinates, average identity and length. [file 1471-2164-13-531-S4.png]

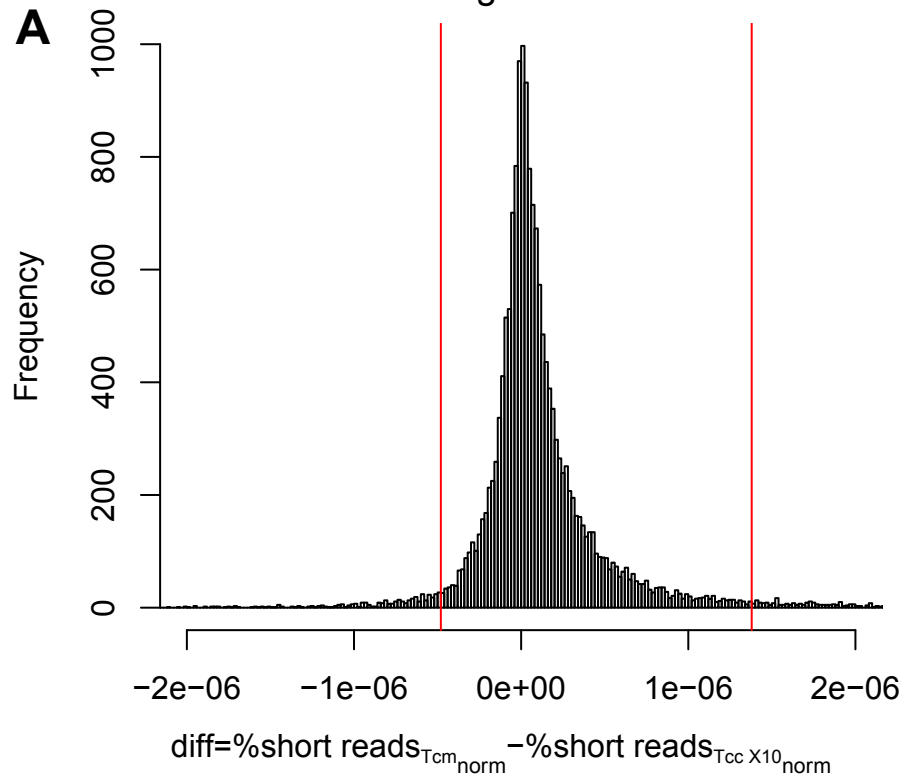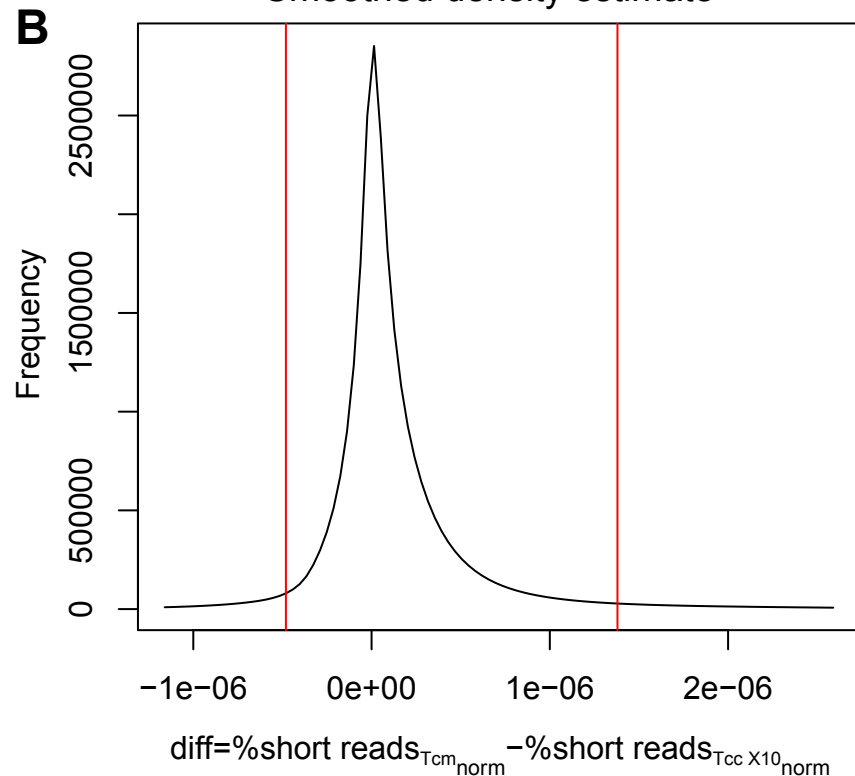

Supplement: Additional file 6 — Table S2. Ratio of non-synonymous and synonymous nucleotide substitutions. Description: Orthologous gene pairs between T. c. marinkellei and T. c. cruzi CL Brener displaying elevated dN/dS (> 1.1). The yn00 program was used to calculate dN and dS. [file 1471-2164-13-531-S6.pdf]

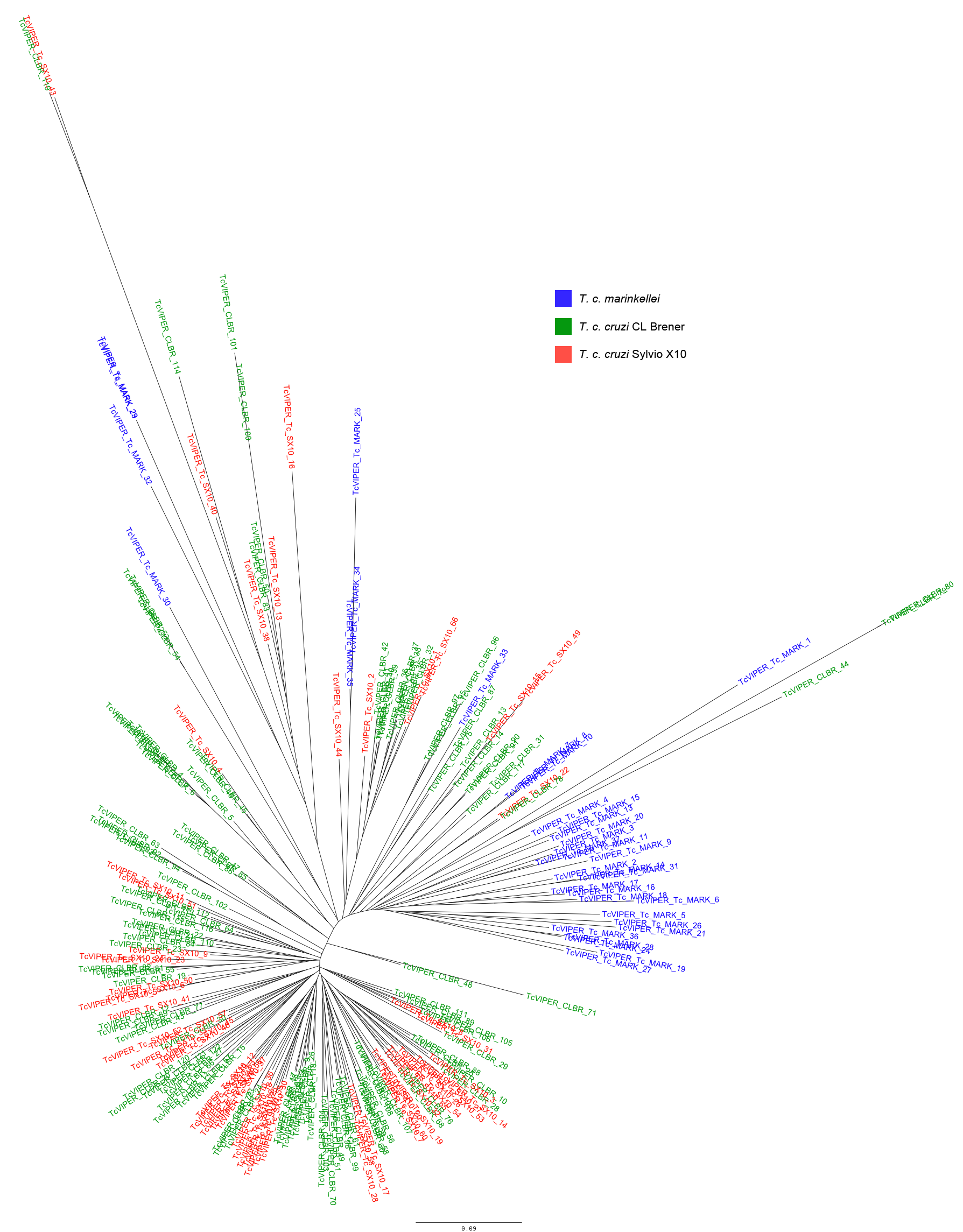

Supplement: Additional file 7 — Figure S5. Disruption of sequence co-linearity. Description: Disruption of chromosomal co-linearity between T. c. marinkellei and T. c. cruzi CL Brener non-Esmeraldo-like (A) as well as between T. c. cruzi Sylvio X10 and T. c. cruzi CL Brener non-Esmeraldo-like (B). Black chromosomes prefixed with ‘Chr’ represent sequences from Tc CL Brener whereas white chromosomes prefixed ‘contig’ represent sequences from Tcm and Tcc X10 assemblies. Alignments were generated using the promer software (Kurtz et al., 2004). Chromosomal stretches marked with green color represent gaps in the assembly. Only gaps larger than 5 kb are shown. The most outer numbers are sequence identifiers. [file 1471-2164-13-531-S7.png]
