# Supplementary material for: Comparative genomic analysis of human infective Trypanosoma cruzi lineages with the bat-restricted subspecies T. cruzi marinkellei
Source: BMC Genomics. 2012 Oct 5;13:531. doi: 10.1186/1471-2164-13-531 (PMC3507753; doi:10.1186/1471-2164-13-531)
Supplement: Additional file 5 — Figure S4. Maxicircle phylogenetic tree. Description: Maximum likelihood phylogenetic tree of the maxicircle sequences from T. c. marinkellei, T. c. cruzi Sylvio X10, T. c. cruzi CL Brener, T. c. cruzi Esmeraldo using T. brucei and L. tarentolae as outgroups. The full maxicircle sequences were aligned with ClustalW v2.1 and the subsequent alignment was filtered using Gblocks (default settings). The tree was inferred using MEGA v5.1 from 13,731 (49%) alignment positions. [file 1471-2164-13-531-S5.pdf]

A

Tc Sylvio X10

0.8 kb

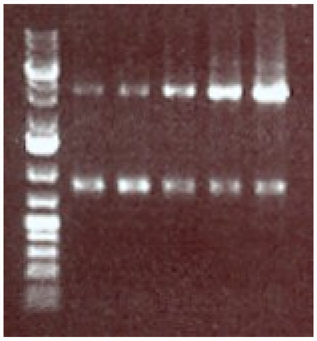

1 2 3 4 5 6

5' gagcatgtcactcgctacca 3'
5' aatggtgccgtgatacacaa 3'

1: Fermentas O'RangeRuler 100 bp+500 bp DNA Ladder
2: 50.0 °C (Annealing temperature)
3: 50.6 °C (Annealing temperature)
4: 51.7 °C (Annealing temperature)
5: 53.5 °C (Annealing temperature)
6: 56.0 °C (Annealing temperature)

B

Tcm

4 kb

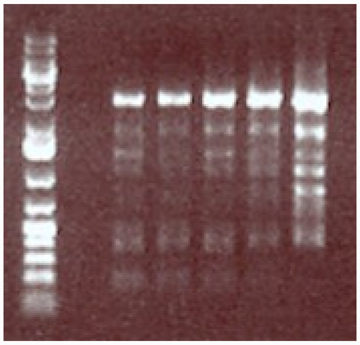

1 2 3 4 5 6 7

5' gtttctacgcgggtgtcatt 3'
5' gtttaatgtggggcagcagt 3'

1: Fermentas O'RangeRuler 100 bp+500 bp DNA Ladder
2: Empty
3: 50.0 °C (Annealing temperature)
4: 50.6 °C (Annealing temperature)
5: 51.7 °C (Annealing temperature)
6: 53.5 °C (Annealing temperature)
7: 56.0 °C (Annealing temperature)

C

Tcm

0.8 kb

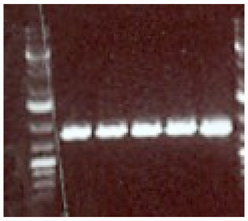

1 2 3 4 5 6

5' ccataaccctgacccaacac 3'
5' accgcgtcaagttgaagaat 3'

1: Fermentas O'RangeRuler 100 bp+500 bp DNA Ladder
2: 50.0 °C (Annealing temperature)
3: 50.6 °C (Annealing temperature)
4: 51.7 °C (Annealing temperature)
5: 53.5 °C (Annealing temperature)
6: 56.0 °C (Annealing temperature)

D

Tcm

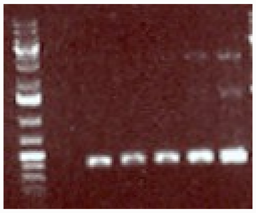

5' atggcgaaagctgcatgtat 3'
5' tgaattaagttctccagctctcc 3'

1: Fermentas O'RangeRuler 100 bp+500 bp DNA Ladder
2: Empty
3: 50.0 °C (Annealing temperature)
4: 50.6 °C (Annealing temperature)
5: 51.7 °C (Annealing temperature)
6: 53.5 °C (Annealing temperature)
7: 56.0 °C (Annealing temperature)

E

Tc Sylvio X10

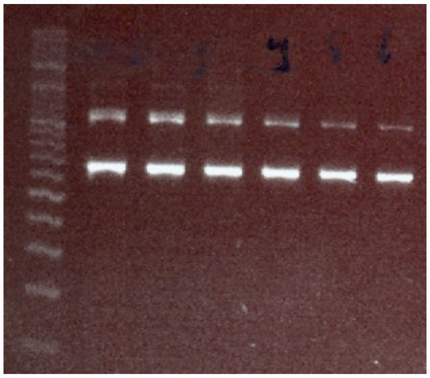

5' ggtaacgccggctataagaa 3'
5' gtacgcaaaaaccggaccta 3'

1: Fermentas O'RangeRuler 100 bp+500 bp DNA Ladder
2: 50.0 °C (Annealing temperature)
3: 50.6 °C (Annealing temperature)
4: 51.7 °C (Annealing temperature)
5: 53.5 °C (Annealing temperature)
6: 56.0 °C (Annealing temperature)
7: 59.2 °C (Annealing temperature)

F

Tc CL Brener

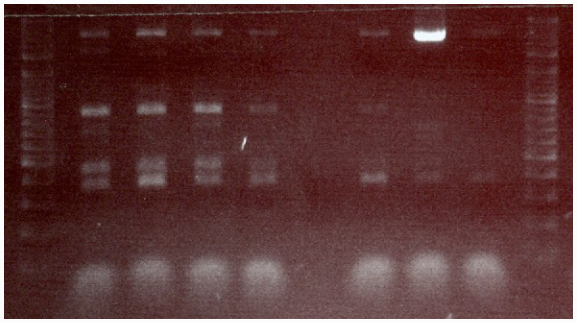

5' gtcctgagtgtctgtggcttc 3'
5' tgtacggtttgatgtacgatgg 3'

1: Fermentas O'RangeRuler 100 bp+500 bp DNA Ladder
2: 50.0 °C (Annealing temperature)
3: 50.6 °C (Annealing temperature)
4: 51.7 °C (Annealing temperature)
5: 53.5 °C (Annealing temperature)
6: 56.0 °C (Annealing temperature)
7: 59.2 °C (Annealing temperature)
8: 62.9 °C (Annealing temperature)
